# Nanomachine Networks: Functional All-Enzyme Hydrogels from Photochemical Cross-Linking of Glucose Oxidase

**DOI:** 10.1021/acs.biomac.4c01519

**Published:** 2025-01-23

**Authors:** Harrison Laurent, David J. Brockwell, Lorna Dougan

**Affiliations:** 1 School of Physics and Astronomy, University of Leeds, Leeds LS2 9JT, U.K.; 2 Astbury Centre for Structural Molecular Biology, Faculty of Biological Sciences, University of Leeds, Leeds LS2 9JT, U.K.

## Abstract

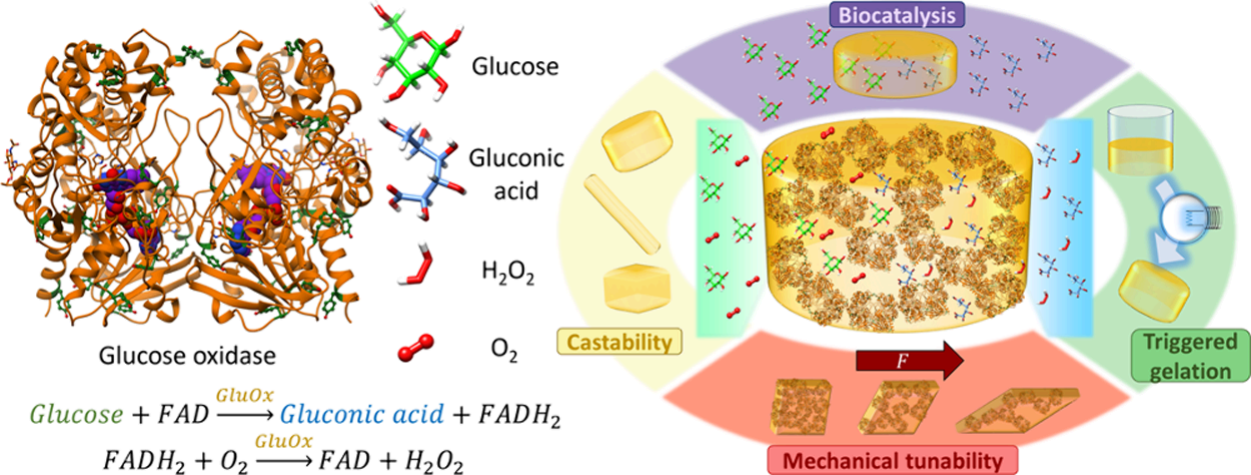

Enzymes are attractive as catalysts due to their specificity and biocompatibility; however, their use in industrial and biomedical applications is limited by stability. Here, we present a facile approach for enzyme immobilization within “all-enzyme” hydrogels by forming photochemical covalent cross-links between the enzyme glucose oxidase. We demonstrate that the mechanical properties of the enzyme hydrogel can be tuned with enzyme concentration and the data suggests that the dimeric nature of glucose oxidase results in unusual gel formation behavior which suggests a degree of forced induced dimer dissociation and unfolding. We confirm and quantify the enzyme activity of the hydrogel using the Trinder assay and a 1D modeling approach and show that 50% enzymatic activity is retained upon hydrogel formation. These observed effects may be due to the forces experienced by the individual nanoscale enzymes during mesoscale network formation. We have therefore demonstrated that photochemical cross-linking can be readily employed to produce functional all-enzyme glucose oxidase hydrogels with easily tunable mechanical properties and specific catalytic activity. This approach provides enormous potential for producing biocatalytic materials with tunable mechanical properties, responsive biological functionality and high volumetric productivity which may inform the future design of biomedical devices with enhanced sensitivity and activity.

## Introduction

Enzymes are a class of protein capable of catalyzing chemical reactions by binding to their compatible substrate(s).^1^ As catalysts, they show desirable qualities over conventionally used chemical catalysts, such as platinum, palladium, and rhodium.^2,3^ These include high substrate specificity, thereby avoiding unwanted side-reactions, good biocompatibility and therefore nontoxicity, and mild reaction conditions. While clearly useful as nanomachines, the practical application of enzyme solutions for catalysis is limited by their stability, as enzymes in solution will gradually denature/aggregate over time and hence lose their activity. This also hinders large-scale production of enzyme-catalyzed chemical products, as extraction of the enzyme is relatively complex and time-consuming. One potential method of overcoming these limitations is to immobilize enzymes onto a surface or within a hydrogel matrix.^4^ This hinders aggregation by limiting the ability of individual enzymes to associate, promotes enzymatic activity by limiting conformational changes, and improves extraction of the enzyme as immobilization within or onto a much larger material means they may be retrieved.^5^ Immobilization of enzymes also has the added advantage that reactions can be rapidly terminated without inactivation of the enzyme by simply removing the immobilized enzyme from the reaction medium. These attractive properties mean that enzyme immobilization has been well researched, including onto nanoparticles,^6,7^ which allows the existing nanoparticle functionalities, such as stimuli responsiveness, catalytic activity, and tumor uptake, to be complemented with enzymatic activity, within cross-linked enzyme crystals/aggregates^8,9^ and within hydrogels through either physical embedding or covalent cross-linking.^4^

Enzyme immobilization within hydrogels, pioneered by Reetz et al.,^10^ is particularly attractive for biomedical applications due to their host of existing favorable properties,^11,12^ including water retention, biocompatibility, the similarity of the network structure to the extracellular matrix, adhesion, mechanical tunability, and responsiveness to external stimuli such as temperature or pH. Hydrogels have therefore found extensive applications in drug delivery,^13,14^ tissue engineering,^15^ and wound healing.^16,17^ Several classes of enzyme have been immobilized in various hydrogels for the production of functional biocompatible materials, including lipase,^18,19^ urease,^20,21^ and glucose oxidase.^22−24^ Enzyme immobilization within hydrogels is achieved either through physical entrapment^5,25,26^ or covalent linking of the enzyme to the network.^5,22,27^ Physical entrapment is attractive as the enzyme requires no prior modification or functionalization and can often be performed using a “one-pot” method. However, there are challenges including achieving an optimal network mesh size, which is sufficiently small to prevent enzyme leakage from the network while sufficiently large that diffusion of substrates/products to and from the enzyme is unhindered.^28^ Covalent immobilization overcomes the issue of enzyme leakage, allowing larger mesh-size networks to be exploited, but requires enzyme modification/functionalization. In both cases, the choice of hydrogel matrix is important, as those that polymerize through heat or the use of additional chemicals may induce a degree of enzyme inactivation. Enzyme immobilization within a separate hydrogel matrix formed from a different polymer suffers from an inherent limitation: poor volumetric productivity, as the hydrogel itself often shows no catalytic activity, and the inherent challenges of purification and scale-up of multicomposite biologically derived or inspired systems.^29,30^ A highly desirable solution is therefore to exploit the enzyme as both the building block to form the structural scaffold of the hydrogel and to provide catalytic functionality, to form “all-enzyme” hydrogels. To date, two primary approaches have been explored to achieve this goal. In both instances, the enzyme is functionalized with additional chemical groups, which allow their self-association into a hydrogel matrix. The first, pioneered by Niemeyer et al., employs a SpyTag/SpyCatcher chemistry to spontaneously form covalent chemical bonds between enzymes and has found several innovative applications in flow biocatalysis.^31,32^ The second, pioneered by Banta et al., uses grafted α-helical domains, which self-associate to form the hydrogel and have been successfully employed as biobatteries.^33,34^

**Figure 1:**
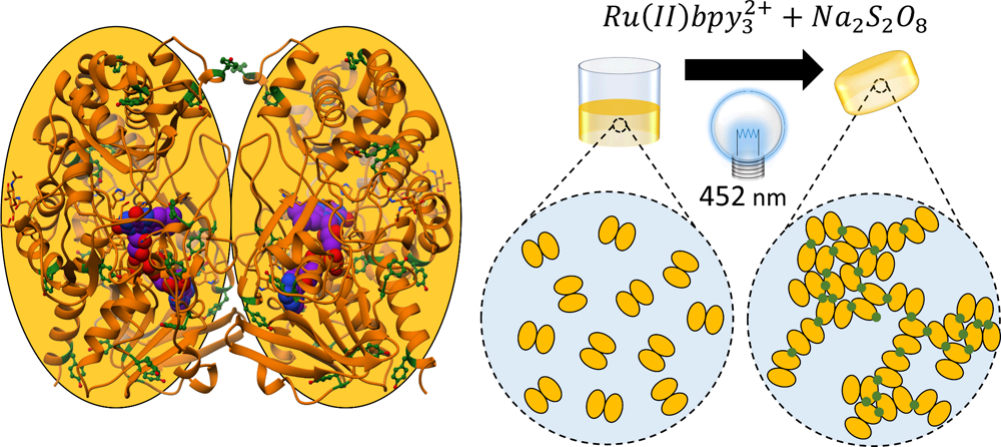
Cartoon representation of the dimeric glucose oxidase from *Penicillium amagasakiense*. (left) (PDB: 1GPE, used as no dimeric structure of GluOx from *Aspergillus niger* currently exists in the PDB) Ribbon structure shown in orange, FAD cofactor shown in purple, and Tyr residues shown in dark green. The rough elliptical shape of each monomer is also shown. Formation mechanism of the all-enzyme hydrogel from glucose oxidase (right) in the presence of sodium persulfate (Na_2_S_2_O_8_) and ruthenium(II) tris-pipyridyl dication (Ru(II)bpy_3_^2+^) and photoactivation at 452 nm. Glucose oxidase dimers are shown as paired yellow ellipses, with formed covalent Tyr-Tyr cross-links shown in green.

In this work, we present a new facile approach for achieving all-enzyme hydrogels. We employ photochemical covalent cross-linking of surface-exposed tyrosine residues on a folded enzyme to form a self-supporting and biologically functional hydrogel. The specific photoactivation ensures the preservation of the folded structure of glucose oxidase (GluOx), as depicted in Figure 1, rather than the more common approach of heat or pH-induced protein gelation, which leads to partial protein denaturation and loss of functionality. We have previously used this photoactivated cross-linking method to form hydrogels from several monomeric globular proteins, including bovine serum albumin (BSA),^35−38^ maltose binding protein from *E. coli* (MBP),^39,40^ protein L,^41^ and the immunoglobin-like domain I27.^42^ Using both experimental and modeling approaches, we have investigated how key parameters, such as cross-linking reaction rate,^35,37,43^ protein concentration,^35^ protein unfolding,^36,37^ ligand binding,^39,40^ and the density and location of cross-linking sites,^44,45^ affect the structural evolution and mechanical properties of the protein hydrogels. In general, we have shown that increasing the cross-linking reaction rate results in rapidly forming sparsely packed gels with lower fractal dimensions and greater shear modulus as one moves from a reaction-limited to a diffusion-limited regime. Increasing protein concentration, ligand binding, and protein unfolding was also shown to increase gel shear modulus, while the fractal dimension was shown to increase as a consequence of allowing protein unfolding or increased solvent accessibility of cross-linking sites. Previous studies on all-enzyme hydrogels using SpyTag/SpyCatcher chemistry have also shown a positive correlation between cross-link density and gel shear modulus.^31,46^ Hydrogel preparation by photochemical covalent cross-linking is advantageous due to its rapid cross-linking time (on the order of minutes), providing a simple method to tune mechanical properties of the resulting hydrogel by modifying the protein concentration or intensity of light used to induce cross-linking^35,37^ and requires no prior modification to the protein so long as it already contains a sufficient number of surface-exposed tyrosine residues.

Glucose oxidase (EC number 1.1.3.4) is an oxidoreductase enzyme, existing as a homodimer in its functional state with each monomer containing a flavin adenine dinucleotide (FAD) cofactor.^47^ It catalyzes β-d-glucose to d-glucono-δ-lactone, which spontaneously hydrolyzes to d-gluconic acid and hydrogen peroxide in the presence of molecular oxygen through a double displacement or “ping-pong” mechanism. This reaction is carried out with remarkable efficiency, with enzymatic efficiency *k*_cat_/*K*_*M*_ values reported up to 38,000 M^–1^ s^–1^ for glucose oxidase from *A. niger* at pH 6.5 and 70 °C.^48^ This property, as well as its commercial availability and breadth of previous literature, makes it an attractive choice for this initial study of photochemically cross-linked all-enzyme hydrogels. As glucose is a compatible substrate for this enzyme, glucose oxidase has been particularly well researched for potential applications in diabetes monitoring and treatment,^49^ including in biosensing,^23,25,27,50^ insulin regulation,^14,51^ treatment of diabetic wounds,^16,17^ and periodontitis.^52^ It has also found applications in cancer therapy,^6^ nanoparticle synthesis,^22^ induction of hypoxia for biological research,^53^ biocatalysis,^54^ and biobatteries^26^ and has EU approval as a food additive.^55^

In this work, we demonstrate that photochemical cross-linking can be employed to produce functional all-enzyme glucose oxidase hydrogels with easily tunable mechanical properties that retain ∼50% of catalytic activity. We suggest that these simply formed materials can serve as a base for biomedical devices with potentially greatly enhanced sensitivity and activity.

## Materials and Methods

### Materials

Glucose oxidase type X-S from *A. niger* (GluOx), peroxidase from horseradish (EC: 1.11.1.7), o-dianisidine dihydrochloride, tris(2,2′- bipyridyl)dichlororuthenium(II) hexahydrate (Ru(BiPy)_3_), sodium persulfate (NaPS), sodium phosphate dibasic, and sodium phosphate monobasic were purchased from Sigma-Aldrich and used without further purification.

### Hydrogel Precursor Preparation

Hydrogels were prepared using a previously described method.^35,36^ All components were suspended in 25 mM sodium phosphate buffer (NaPB) at pH 7.4 prepared from aqueous sodium phosphate monobasic and sodium phosphate dibasic. Briefly, a 4× concentrated regent stock containing 200 mM NaPS and 0.4 mM Ru(BiPy)_3_ in NaPB was prepared. An enzyme stock solution of 14–35 mg of glucose oxidase gently suspended in 150 μL of NaPB in 500 μL eppendorf was also prepared. The resultant solution was then centrifuged at 150,000g for 1 min to remove any aggregates and the supernatant containing free enzyme removed. This results in a stock solution of free enzyme at 133% of the final concentration desired in the hydrogel. The reagent stock and the enzyme stock were then mixed in a 1:3 volume ratio to achieve the final hydrogel precursor. This method ensures that the cross-linking agent NaPS was always in molar excess of tyrosine (the most extreme estimate assuming the highest employed enzyme concentration of 108 mg/mL and assuming all tyrosine residues are solvent exposed suggests a tyrosine concentration of 46 mM). This solution can then be illuminated at 452 nm to form covalent bonds between surface-exposed tyrosine residues on neighboring enzymes.^56^ Using the only known dimeric structure for glucose oxidase (from *P. amagasakiense*, PDB code 1GPE), we estimate the total number of surface-exposed Tyr residues to be between 13 and 16, comprising a total solvent-accessible surface area of ∼760–900 Å^2^. This is described in more detail in the Discussion section. The concentration of the enzyme stock solution was measured by the absorbance of a 200–500 × diluted sample at 280 nm on a Shimadzu UV-1900i UV–vis spectrophotometer and applying the Beer–Lambert law. Using the sequence of GluOx derived from PDB code 1CF3, the molar extinction coefficient and molar mass of GluOx were taken to be 190710 M^–1^ cm^–1^ and 126546.8 g mol^–1^ respectively. Such a large dilution is required as the apparatus can only measure reliably absorbance values up to ∼1; however, such large dilution necessarily results in large errors in measured concentrations. To account for this, the measured concentrations [GluOx] of the enzyme stock solutions (scaled by 75% to account for the enzyme concentration in the resultant hydrogel) were plotted as a function of initially added mass *m*_GluOx_ and fitted to a function of the form shown in eq 1. This predicts fitting parameters of α = 0.002516 and β = 0.23869 and now allows the enzyme concentration in the hydrogel to be more reliably predicted from the mass of the initially added enzyme. This process is shown in Supplementary Figure S1.
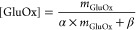
1

### Rheology Measurements

**Figure 2:**
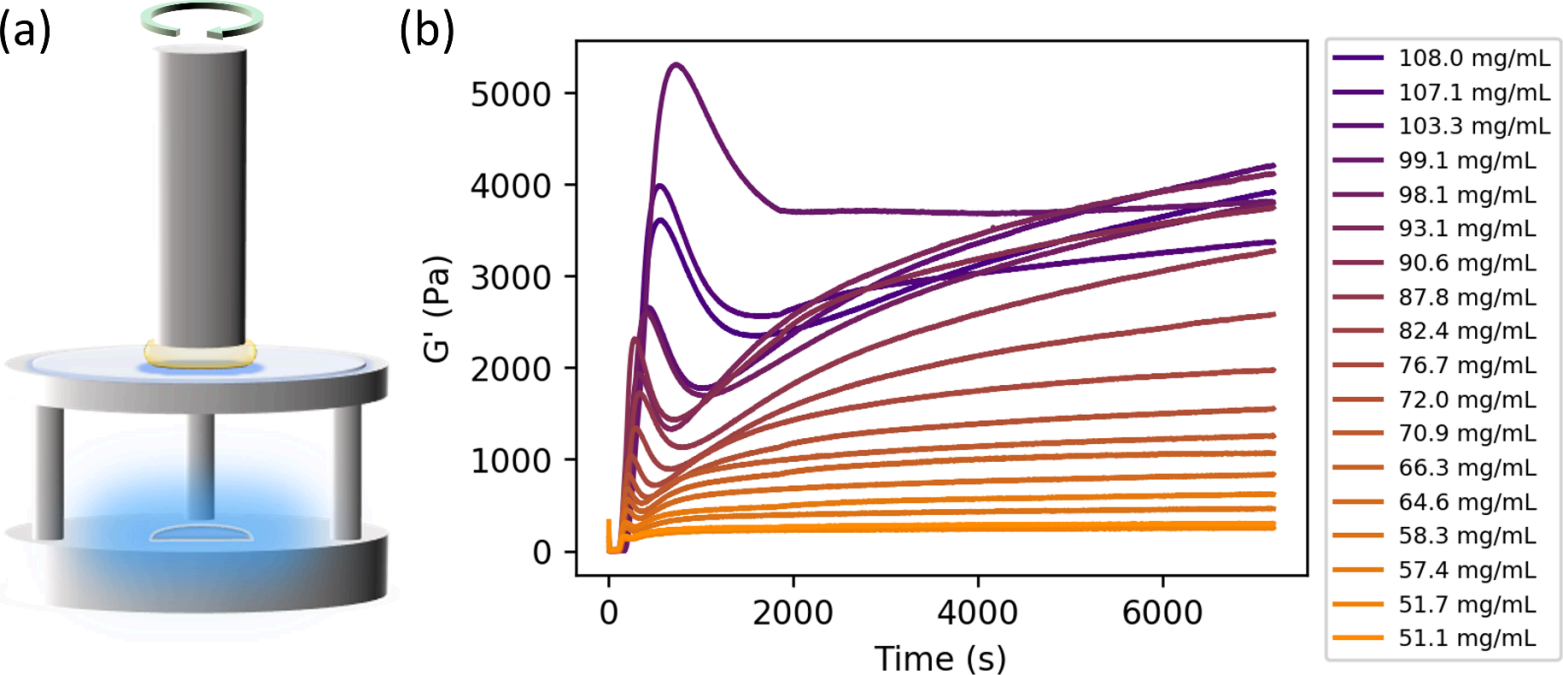
(a) Schematic of a custom blue LED rig used for *in situ* gelation rheology measurements on a stress-controlled Anton Paar 302 rheometer. (b) Time-resolved storage modulus *G*′ as measured by oscillatory shear rheology of GluOx hydrogels at varying concentrations. The concentrations studied are colored using an orange–purple color scheme described in the figure legend. Quoted concentrations correspond to enzyme volume fractions varying between 3.8 and 8.0% assuming an enzyme density of 1.35 g/cm^3^.^60^ Corresponding frequency sweeps taken at *t* = 7200 *s* are available in Supplementary Figure S6.

Rheology measurements were performed on an Anton Paar MCR 302 stress-controlled rheometer (Anton Paar, GmbH, Austria) using an 8 mm parallel plate geometry to minimize the sample volume. The parallel plate configuration was used due to the custom light rig employed to trigger *in situ* gelation. This comprises a blue LED (peak emission at 452 nm) physically isolated below an acrylic stage onto which 40 μL of the gel precursor solution was placed, resulting in a gap height of ∼0.72 mm, as shown in Figure 2a. A parallel plate geometry therefore allows for a uniform light field through the entire gel. The corresponding light intensity through the gel was measured to be 35.1 mW/cm^2^. Time sweep data was taken using a frequency of 1 Hz and strain of 0.5%, with data recorded every 3 s for a total time of 7200 s.

### Enzymatic Activity

Enzymatic activity of free GluOx was calculated using the procedure pioneered by Phil Trinder^57^ in a 1 cm path length-matched quartz cuvettes. Briefly, a reaction medium was prepared in NaPB containing 1 nM GluOx, 200 nM peroxidase from horseradish, and 160 μM o-dianisidine dihydrochloride. Upon addition of 5–100 mM glucose, GluOx begins to catalyze the reaction of glucose to gluconic acid and H_2_O_2_. The peroxidase in turn then catalyzes the oxidation of o-dianisidine by H_2_O_2_, which turns brown. This process was followed on a Shimadzu UV-1900i UV–vis spectrophotometer, which simultaneously monitors the absorption at 460 nm of the sample, using a molar absorption coefficient for oxidized o-dianisidine of 1.13 × 10^4^ M^–1^ cm^–1^,^58^ and a corresponding reaction medium control, which does not contain GluOx. All data was obtained at 25 °C. The initial reaction rates *V*_0_ are then calculated over the first 100 s as a function of glucose concentration, and the resulting data were analyzed using Michaelis–Menten kinetics. To apply this procedure for GluOx as a hydrogel, GluOx hydrogels were formed by injecting a 90 mg/mL precursor solution into a 2 mm-diameter PTFE tube treated with Sigmacote and illuminated for 15 min using a white lamp at ∼10 cm. The corresponding light intensity using this method was measured as 125 mW/cm^2^. The resultant gels were then extruded out of the PTFE tube into 15 mL of NaPB and weighed. To remove any unbound enzyme, as well any unreacted chemicals present in the reagent stock, the gels were shaken on a slowly oscillating tilt table for >30 min, before being removed and replaced into 15 mL of fresh NaPB. This procedure was repeated for three washing cycles, with the last cycle being left in the solution for 24 h. To validate that all enzyme and additional chemicals had been successfully washed out of the gel, the absorption spectra of each wash buffer were taken and compared with controls of free GluOx in NaPB and unused NaPB. This methodology shows successful washing following this procedure, as shown in the Supplementary Figure S2. The final successfully cast gel (image shown in Supplementary Figure S3) was then weighed to calculate the swelling ratio and was then cut to ∼1 cm in size and each reweighed. All used gels were between 35 and 50 mg. The formed 1 cm gels were used in place of the 1 nM GluOx in the procedure described above, placing the gel at the bottom of the quartz cuvette. The swelling ratio was calculated to be 54.3 ± 0.9% according to eq 2:
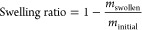
2

### Circular Dichroism

Far-UV circular dichroism (CD) spectra were acquired on a Chirascan plus CD spectrometer (Applied PhotoPhysics) with a bandwidth of 2 nm, a step size of 1 nm, and a commercially available cuvette (Hellma) with a path length of 10 μm at 25 °C over ∼13 h. The signal at 222 nm, corresponding to alpha helices,^59^ was monitored as a measure of folded enzyme concentration. The resultant data were normalized to the value taken for the gel precursor solution, and this value was taken to be 100% folded.

## Results

### Hydrogel Mechanics Using Time-Resolved Oscillatory Shear Rheology

Gelation was triggered by the LED after 1 min of data acquisition, with the LED remaining active during the entire remaining data acquisition period. Silicon oil was added around the sample to prevent evaporation. The resulting data for the storage modulus *G*′ of the GluOx hydrogels, measured using oscillatory shear rheology as a function of time for a range of GluOx concentrations, is shown in Figure 2b. The signal prior to LED illumination (0–60s) shows noisy data in both storage modulus *G*′ and loss modulus *G*″ (shown in Supplementary Figure S4), with values for all data sets between ∼0 and 15 Pa. This noise consequently means that the ratio of *G*″ to *G*′, tan δ, shows a large variation within the allowed range of the instrument (2 × 10^4^). This is likely due to the 8 mm parallel plate setup, whose relatively small diameter will likely result in decreased SNR. Averaging *G*′ and *G*″ data over the first 60 s (*n* = 20) and calculating tan δ manually this way suggests values all lie around ∼1, consistent with a viscoelastic fluid. These data are shown in Supporting Information Figure S5.

**Figure 3:**
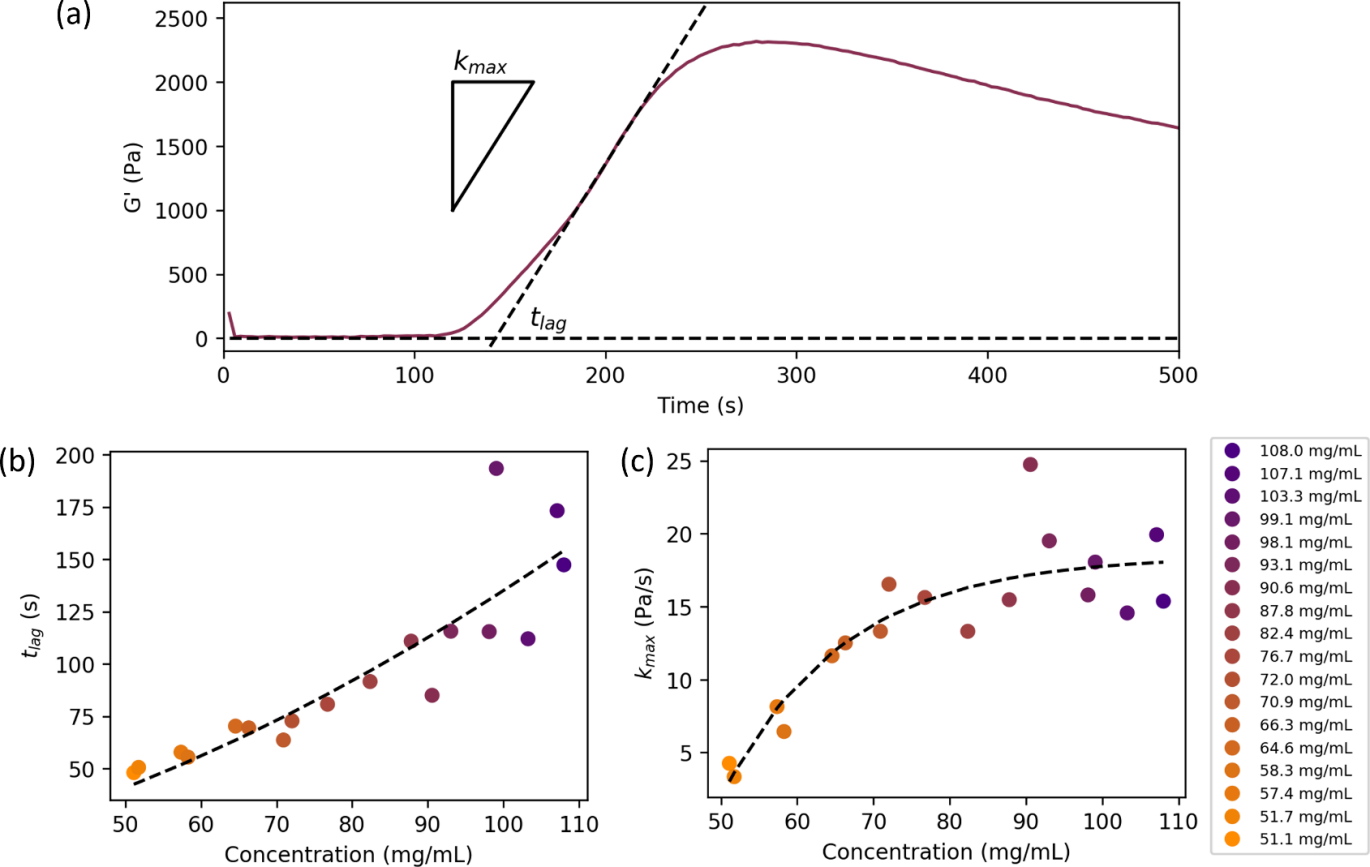
(a) Illustration of extraction of gel formation rate *k*_max_ and lag time *t*_lag_ using 90.6 mg/mL data. (b) Calculated lag time *t*_lag_ of gel formation determined through linear fits shown in Figure S2 as a function of concentration of GluOx in hydrogels. (c) Calculated gel formation rate *k*_max_ determined through linear fits shown in Figure S6 of the Supporting Information as a function of concentration of GluOx in hydrogels. The concentrations studied are colored using an orange–purple color scheme described in the figure legend. Empirical function fit of forms described in Supplementary eqs S2 and S3 as guides to the eye. The recorded data show occasional significant discrepancies, which may arise from the intrinsic variability of the linear fitting method employed due to the user-defined fitting range and the difficulty in accurately determining the enzyme concentration as discussed in the Materials and Methods section.

As the percolated gel network begins to form due to LED illumination, the *G*′ data begin to increase sharply toward a peak. Consequently, the measured values of tan δ then become much less noisy due to the appearance of larger, and more accurately measurable, storage and loss moduli, settling between ∼0.05 < tan δ < 0.125, consistent with a gel-like behavior. Fitting a linear function to this initial increase, as demonstrated in Figure 3a, allows for calculation of the maximum formation rate *k*_max_, and lag time *t*_lag_ (time at which the function predicted by the linear fit to the gel formation is equal to 0). These (Figure 3b,c) show that the *t*_lag_ increases with concentration over the full measured range, and the *k*_max_ increases with concentration, before plateauing beyond an enzyme concentration of ∼80 mg/mL. The behavior of *t*_lag_ with concentration is highly unusual and is discussed further in the Discussion section.

**Figure 4:**
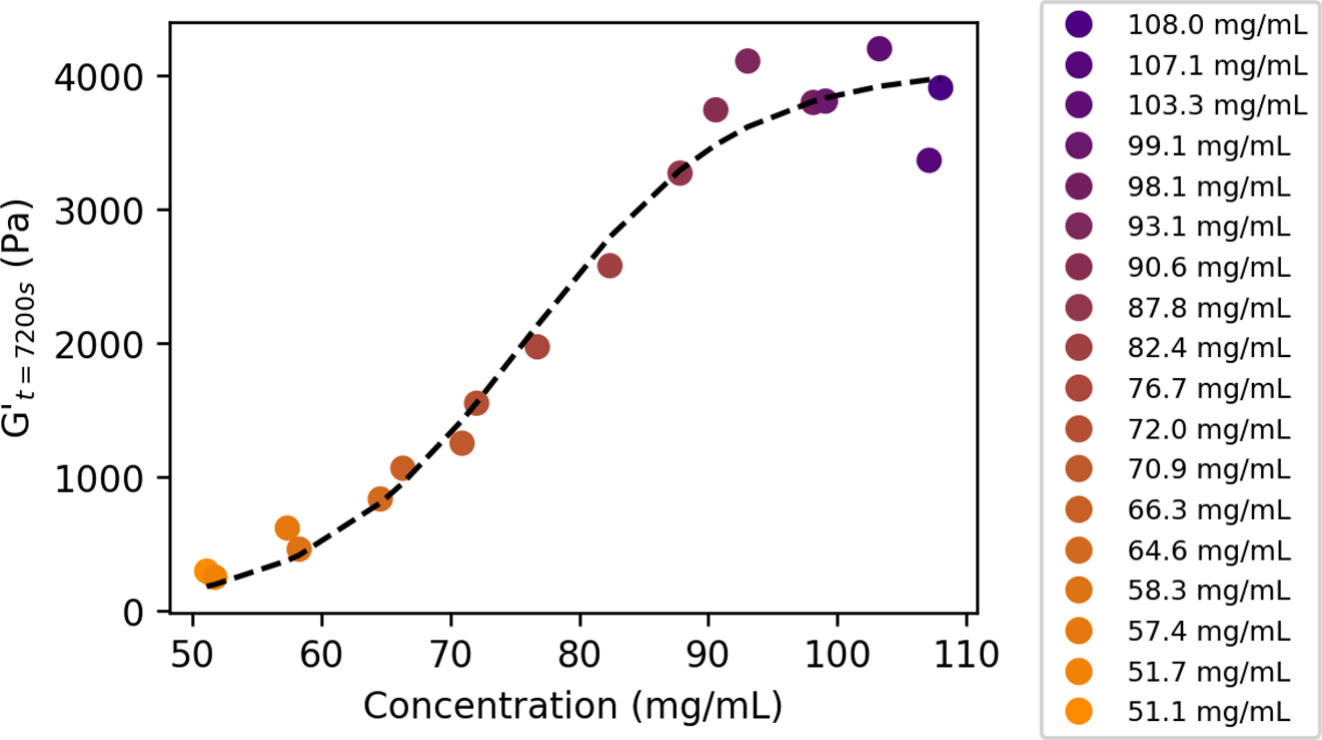
Value of storage modulus *G*′ at 7200 s for GluOx hydrogels. Sigmoidal function was fitted to data as a guide to the eye. The concentrations studied are colored using an orange–purple color scheme described in the figure legend.

We then observed a complex relaxation profile with time. Following the formation of the hydrogel network to a peak storage modulus, the gel then weakens and *G*′ reduces to a local minimum. This is then followed by a gradual recovery of *G*′ over the remainder of the experimental time frame. The final recorded value of *G*′ (*t* = 7200 *s*) for each concentration is shown in Figure 4. Here, we observe a gradual increase as a function of concentration until a plateau past ∼90 mg/mL. This plateau suggests that the maximum energy storing capacity of the network backbone when formed from this particular enzyme building block is reached around this concentration. Any additional enzyme supplied to the system at this point likely joins the network in such a way that it does not contribute to the bulk rheological properties, but likely would contribute to the overall enzymatic activity of the hydrogel.

### Folded Fraction of Enzyme in the Hydrogel

**Figure 5:**
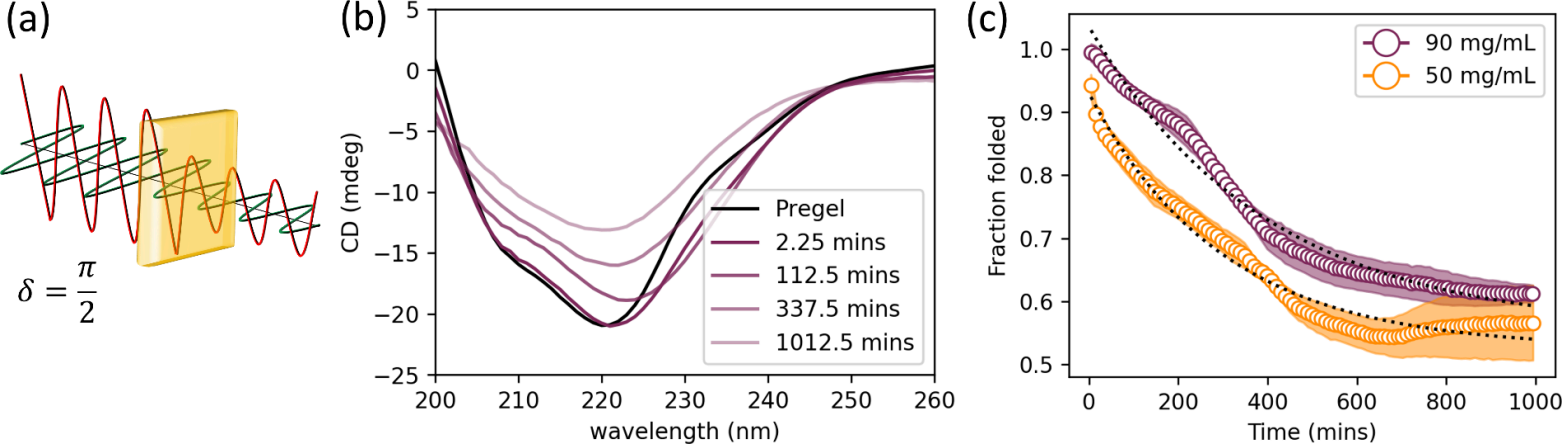
(a) Origin of CD. Left-handed and right-handed circularly polarized lights (LHCP and RHCP) occur when the horizontally and vertically polarized components of light are separated by a phase difference of  respectively. When this is incident on intrinsically chiral structures, such as those found in proteins, it will be absorbed with a dependence on the wavelength. The differing absorbance of LHCP and RCHP results in light with an overall elliptical polarization, the ellipticity of which is measured in millidegrees (mdeg).^59^ (b) CD spectra at various time points from 90 mg/mL data to illustrate how gradual unfolding of the enzyme due to gelation forces causes a change in the signal. Pregel solution is colored in black; the color corresponding to the 90 mg/mL gel in previous figures is used or the formed hydrogel, with increasing transparency used to represent increased time. (c) Folded fraction of enzyme as a function of time determined by normalized CD signal of 90 and 50 mg/mL gel at 222 nm to signal at 222 nm in pregel solutions. Data points are shown as open circles, colored to correspond to the 90 and 50 mg/mL concentrations employed in previous figures, to improve clarity of exponential fit shown as dotted black line. Standard error shown with accordingly colored error ribbons for two repeats at each concentration.

CD was used to monitor the proportion of folded protein in the pregel solution and immediately after gelation for GluOx concentrations corresponding to 90 and 50 mg/mL. CD monitors secondary structure content and consequently, the degree of folding by monitoring the signal shape and amplitude.^59,61^ While the high enzyme concentrations employed in this study mean that the data can be subject to a larger degree of uncertainty than would be expected for typical solution-state measurements, as shown by the slight variability of data presented in Figure 5c, CD remains a sensible choice as this limitation would also be expected in infrared spectroscopy or nuclear magnetic resonance techniques and does not rely on deuterated buffers. We choose to monitor the signal at 222 nm as this is a strong signal in proteins containing alpha helices, and GluOx comprises ∼36% alpha helical secondary structure.^59,62^ The data are normalized to the signal at 222 nm in the pregel solution, and this was taken as 100% folded. Data show an exponential decay with time toward a final folded fraction in the gel of ∼60% at both 90 and 50 mg/mL. This suggests that a significant proportion of enzyme remains folded post gelation and is likely to remain active. The exponential decay in the proportion of folded protein is described by a time constant of ∼23,800 s and ∼18,000 s for 90 and 50 mg/mL, respectively, when fit with an equation of the form shown in eq 3, where *FF* is folded fraction, *A* is an amplitude of the decay, and *t* is time. At long times, these fits also suggest that the final folded fraction approaches ∼61% for the 90 mg/mL hydrogel and ∼57% for the 50 mg/mL hydrogel. These results are similar to those of previous CD studies on folded protein hydrogels. Hydrogels formed from maltose binding protein, with and without bound maltose, reached a similar final folded fraction of ∼67%; however, in this instance, the time constants were shorter, with values of 4000 and 2900 s, respectively:^39^
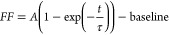
3

This suggests that the rapid gelation induced by this method of photochemical cross-linking results in an applied force exerted on individual enzymes, resulting in some enzyme unfolding.^36^ As a lower concentration necessitates less material to span an equivalent volume to form a percolated gel network, it therefore follows that more force is exerted on each individual enzyme in the 50 mg/mL gel compared with the 90 mg/mL gel. This would lead to a more rapid gelation-induced unfolding, as supported by the lower value of τ in the 50 mg/mL gel and a lower final folded fraction and the large initial decrease in folded fraction immediately following the 5 min gelation. It also suggests that any enzyme unfolding as a result of gelation, as has been observed previously in photochemically cross-linked protein hydrogels, is a comparatively slow process.^35,36,39−41^ An interesting observation is that both concentrations result in a similar final value of the unfolded enzyme. Structural models for photochemically cross-linked protein hydrogels derived by neutron and X-ray scattering^35,36,39^ suggest clusters of folded proteins are connected by regions of unfolded proteins. The CD data suggests that within the concentration range of 50–90 mg/mL, the ratio of folded to unfolded enzyme and, therefore, the ratio of clustered to unclustered enzymes in the hydrogel network may be similar.

### Hydrogel Enzyme Activity Measured with Trinder Assay

**Figure 6:**
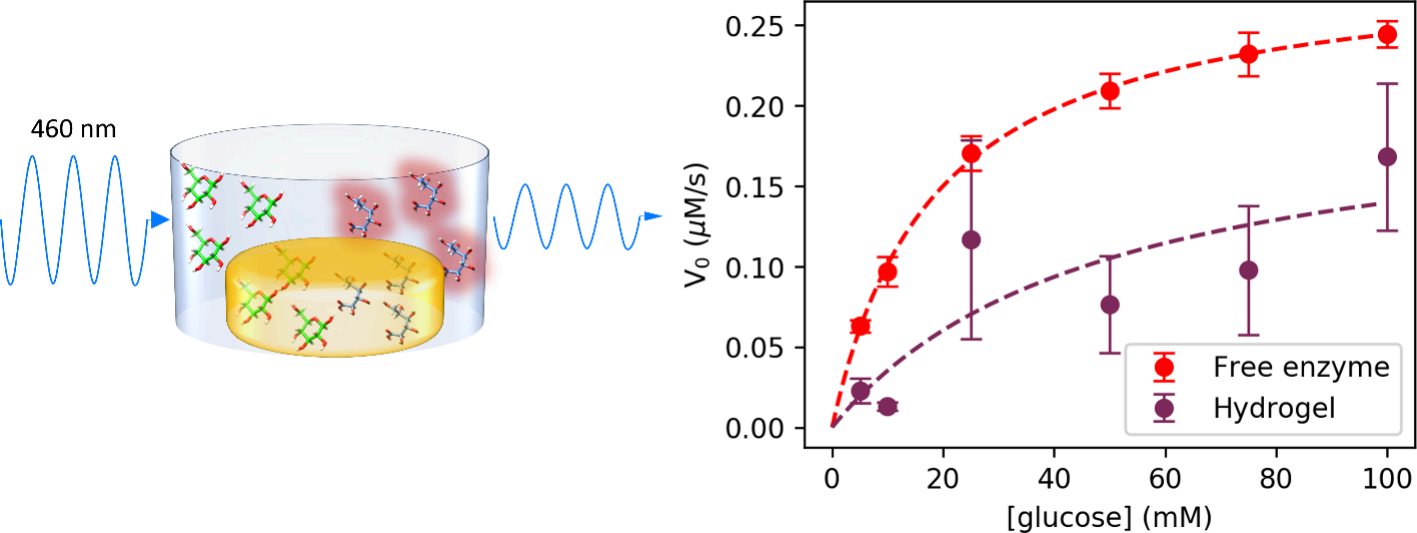
Enzymatic velocities (rate of gluconic acid and hydrogen peroxide production over the first 100 s of data acquisition measured through absorption at 460 nm) as determined using the Trinder assay for free glucose oxidase in the solution (red) and a photoactivated chemically cross-linked all-enzyme hydrogel (colored to correspond to 90 mg/mL data shown in previous figures). Standard errors shown for three repeats at each glucose concentration.

The enzymatic velocities of free GluOx in solution and the GluOx hydrogels measured using the Trinder assay method are listed in Figure 6. The resultant fits to the data using Michaelis–Menten kinetics, described in eq 4 where *V*_0_ is reaction velocity, *V*_max_ is maximum reaction velocity, [glucose] is glucose concentration, and *K*_*M*_ is the Michaelis constant, are shown in Table 1. As the catalysis of glucose to gluconic acid by GluOx proceeds via a double displacement, or ping-pong mechanism, involving both glucose and molecular oxygen, the use of Michaelis–Menten kinetics, which assumes only a single substrate species, is a simplification. However, it has been shown that under the conditions of excess oxygen, it can still be reasonably applied.^63^ The values obtained for the free enzyme are consistent with previous literature values^64^ and are close to the activity stated by the manufacturers (152 U/mg, where 1 U is defined as the catalysis of 1 μmol of substrate per minute). Assaying the hydrogel in place of the free enzyme confirms that the enzyme retains activity after gelation and swelling for 24 h. However, the fitted results shown in Figure 6 suggest that gelation of the enzyme results in a *K*_*M*_ higher than the predicted *K*_*M*_ value and a strong reduction in turnover number *k*_cat_, enzymatic efficiency *k*_cat_/*K*_*M*_, and specific activity. The swelling ratio calculated using eq 2 was used to scale the enzyme concentration in the hydrogel for the calculation of these values by accounting for the additional volume and hence reduced concentration due to buffer uptake by the hydrogel. It is worth mentioning at this point that in the hydrogel state, the observable color change initially occurs in the immediate vicinity of the hydrogel at the bottom of the cuvette and drifts upward into the path of the beam to be detected. This contrasts with a gradual smooth color change throughout the cuvette in the case of the free enzyme. The consequence of this is that the recorded values for enzymatic velocity for the hydrogel reported in Figure 6 show larger error than those of the free enzyme:
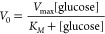
4

**Table 1:** Predicted Values from Michalis-Menten Kinetics for Free GluOx in Solution

**property**	**value (free enzyme)**	**value (Kovačević et al.**(64)***)***	**value (hydrogel)**
*K*_*M*_	18.6 mM	23.19 mM	49.0 mM
*k*_cat_	289.7 s^–1^	130.2 s^–1^	0.143 s^–1^
	15.5 mM^–1^ s^–1^	5.61 mM^–1^ s^–1^	0.0029 mM^–1^ s^–1^
*V*_max_	2.90 × 10^–7^ M/s	Not reported	2.08 × 10^–7^ M/s
specific activity	137.4 U/mg	Not reported	67.7 U/mg

## Discussion

### Gel Formation Rate and Lag Time

Previous literature and theory would suggest that increasing the concentration of GluOx should result in a reduced mean free path between proteins in the solution, and hence, the gelation rate *k*_max_ should increase with increasing concentration. This is observed in other photochemically cross-linked hydrogels, including those based on ionic liquids,^65^ poly(ethylene glycol),^66^ BSA,^35^ and modeling studies.^43^ The data shown in Figure 3c are consistent with this view. However, this argument also suggests that the lag time *t*_lag_ should decrease with increasing enzyme concentration, whereas the data shown in Figure 3b shows the opposite trend, which is empirically fit using eq 5, with fitted results of Amplitude = 1.72 and *x* = 0.049. This is unusual; however, similar effects have been observed in aluminosilicate gels:^67,68^
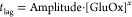
5

In those studies, the authors combine commercially available sodium aluminate/silicate solutions with water and NaOH to form gels through a condensation reaction. The authors explore the gel formation through the gel time (time at which tan δ = 1) by holding two of the three concentrations of their species of interest (Si, Al, or Na) constant while varying the third. In the case of increasing Si, this necessarily involves adding more Na^+^ that is present in the precursor sodium silicate solution. To maintain a constant Na^+^ concentration, the amount of added NaOH must therefore be reduced. This disfavors the decondensation of silicate oligomers into more reactive and mobile monomers by OH^–^, slowing gel formation. Simply put, while increasing Si concentration increases the amount of material available to form the hydrogel, the method by which it is added results in reduced reactivity of the monomer and hence slowed gelation.

Inspired by this observation, we can now consider the GluOx dimer as an equilibrium condition between the functional homodimer and the dissociated monomers, as shown in eq 6:
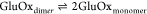
6

We now consider the reactivities of the two species. By aligning the predicted structure for monomeric GluOx from *A. niger* (PDB code: 1CF3), as used in this work, with the only known dimeric structure for glucose oxidase (from *P. amagasakiense*, PDB code 1GPE), we can estimate the number of surface-exposed Tyr residues that can potentially participate in cross-linking reactions and the total solvent assessable area, for GluOx from *A. niger* , as both a dimer and a monomer. Size exclusion chromatography combined with multiangle light scattering (SEC-MALS) was used to verify that the GluOx employed here was indeed dimeric. These data are shown in Supplementary Figure S9. This suggests that in the monomeric form, 23 of the 27 Tyr residues are solvent exposed (SAS > 1 Å^2^), with a total Tyr SAS of ∼1100 Å^2^. In the dimeric form, between 7 and 10 of these residues are buried at the interface and are not available to participate in the cross-linking reaction, resulting in a reduction in total Tyr SAS by between ∼200–340 Å^2^. The volume of the dimer, determined by fitting an ellipsoid to the two structures, is also larger than that of the monomer by a factor of ∼2, resulting in reduced mobility and further reduced reactivity.

As the observed increasing lag time with GluOx concentration is indicative of fewer reactive species with increasing GluOx concentration, it therefore suggests that the initial phase of the GluOx hydrogel formation is dissociation of the dimer into more reactive monomers, likely due to force applied across the dimer interface as the cross-linking reaction begins. This would likely occur more rapidly at lower GluOx concentrations, as the requirement to span a constant volume using less available material necessitates that more force be applied to each individual GluOx enzyme.

### Time-Resolved Enzyme Unfolding

One can also begin to correlate the time constants derived by CD data to the rheological data shown in Figure 2. As described in previous studies,^36^ time-resolved rheology data for photochemically cross-linked protein hydrogels can be described by a single equation of form as presented in eq 7:
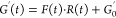
7Here, the time evolution of *G*′ is described by a sigmoidal formation expression *F*(*t*), described in eq 8, and several summed exponential relaxation expressions *R*(*t*), described in eq 9, where *G*_∞_^′^ is the final storage modulus, *t*_0_ is the gel percolation lag time, *C* describes the steepness of the sigmoidal formation factor, *B*_*i*_ are exponential amplitudes, τ_*i*_ are exponential decay constants, and *G*_0_^′^ is the storage modulus of the pregel solution prior to LED illumination:
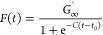
8
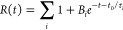
9

**Figure 7:**
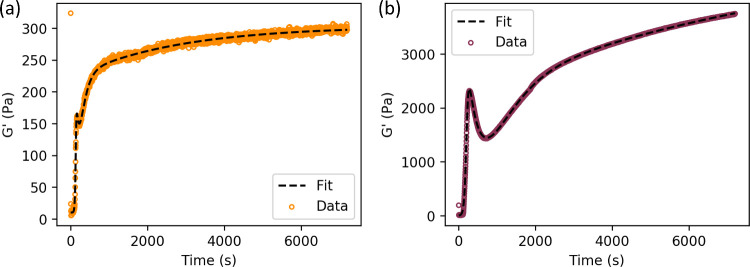
Fitting eq 6 to the storage modulus with time relaxation data for 50 (a) and 90 (b) mg/mL GluOx gels. Data points shown as open circles, colored to correspond to the 90 and 50 mg/mL concentrations employed in previous figures, to improve clarity of exponential fit shown as dotted black line.

Previous studies^36^ have used a single exponential term in *R*(*t*) to describe photochemically cross-linked BSA hydrogels or two exponential terms to describe photochemically cross-linked BSA hydrogels containing dithiothreitol (DTT), which promotes protein unfolding by reducing disulfide bonds, or MBP.^40^ In this work, we find that we require three exponential terms to capture the features of the data presented in Figure 2, two of which must be described by negative exponential amplitudes. To reduce overparametrization, the value for *B*_1_ was fixed at unity. The fitted results for 50 and 90 mg/mL are shown in Figure 7, and full-fitted parameters are reported in the Supplementary Table S2.

Here, we observe that the exponential term in *R*(*t*) with the longest derived time constants has a positive amplitude *B*_*i*_ and hence is describing a process that is leading to gradual gel strengthening by increasing cross-link density. This feature has been previously attributed to protein unfolding^36,40^ and subsequent physical cross-linking between unfolded proteins and in this case was determined to be ∼2700 and ∼11,300 s for 50 and 90 mg/mL gels, respectively. While these show the same qualitative trend of lower time constants at lower concentrations determined through CD measurements in Figure 5c, (∼18,000 and ∼23,700 s for 50 and 90 mg/mL respectively), the values determined through rheology are both lower. This is consistent with previous literature, where it is proposed that the additional strain on the hydrogel imposed by the rheometer is likely to promote protein unfolding and result in a shorter measured decay constant by a factor of ∼2.^36,40^ The different measured values of the time constants from CD and rheology is observed to be larger for the 50 mg/mL sample compared to that of the 90 mg/mL sample.

Another possible explanation for the shorter time constants observed with rheology as opposed to CD is that rheology will only be sensitive to those enzymes in the hydrogel network, which are contributing to the bulk rheological properties (those that lie on the elastic gel backbone if one is to liken the system to colloidal gels^69,70^), whereas CD will consider all enzymes equally. The load-bearing role of enzymes in the hydrogel backbone likely means they will be under the greatest forces in the hydrogel network and will show accelerated unfolding kinetics, described by shorter time constants. This in turn raises the important point that it is well established that the mechanical unfolding of proteins is dependent on the pulling force, pulling direction, and sites from which the pulling force is applied.^71−73^ As the network is made up of many enzymes, covalently bound by their variously distributed exposed tyrosine residues, it is likely that the CD signal arising from enzyme unfolding contains contributions of many unfolding rates. Indeed, it is true that while the data in Figure 5c is reasonably well described by a single exponential decay and this simple analysis gives useful insight, the data are somewhat more variable. Additional validation of the structural transitions underlying the three proposed time constants from rheology and the enzyme unfolding monitored by CD will require future work, using time-resolved scattering techniques.

### Trinder Assay to Quantity Enzyme Hydrogel Functionality

The results of the Trinder assay suggest that immobilizing GluOx in an all-enzyme hydrogel through photochemical cross-linking causes the apparent value of *K*_*M*_ to increase. As *K*_*M*_ is a measure of enzyme–substrate affinity, with low values corresponding to high affinity and *vice versa*,^1^ this suggests that the gelation process reduces the GluOx-glucose affinity. This seems sensible, as the enzymes within a hydrogel are under force,^36,39,74^ as supported by Figure 5. In the case of GluOx, this force is likely to be applied across the dimer interface to some extent, which defines the active site of the enzyme, and would likely reduce affinity. These data also show that the specific activity is reduced from 137.4 to 67.7 U/mg. This would initially suggest that the gelation process inactivates approximately half of the available enzyme; however, it is important at this stage when understanding these results that one considers diffusion limitations. Previous literature data has demonstrated that GluOx covalently immobilized onto silica nanoparticles and entrapped in poly(ethylene glycol) (PEG) hydrogels exhibited strongly reduced *V*_max_ values and increased *K*_*M*_ values as the mesh size of the hydrogels was reduced, and hence, diffusion became increasingly limited.^75^ We can validate this finding further by using a simple 1D diffusive model.

Within this model, we define a 2 cm length (roughly equal to the length of the quartz cuvettes employed in the experimental data), discretized into 100 μm (*d*_slice_) slices. We then allow barrierless diffusion between slices, with the flux of material at each border between each slice *J* defined by a 1D Fickian diffusion. This is described by eq 10 , where Δφ is the difference in the concentration of a given solute between two consecutive slices, and *D* is the diffusion coefficient:
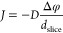
10

**Figure 8:**
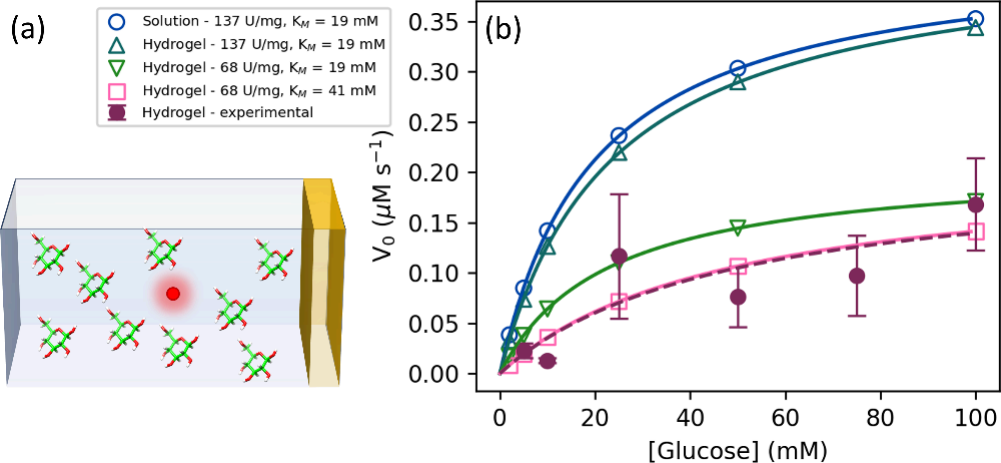
(a) Schematic illustration of starting state of simulation *t*_0_, where hydrogel is localized to the far right of the simulation length (yellow), the glucose is allowed to occupy the remaining length (green), and the signal is monitored in the center of the length (red). (b) Enzymatic velocities as a function of glucose concentration for 1D diffusive models (colored using a blue to green to pink color scheme, with open symbols representing the simulated glucose concentrations and Michaelis–Menten fits shown as solid lines) and experimental data from Figure 6 using enzyme hydrogels initially prepared at 90 mg/mL (colored to correspond to 90 mg/mL data shown in previous figures.

As a first step, we can model a solution-like enzyme catalysis by allowing Michaelis–Menten kinetics to occur over the full length of the simulation (reactants can be catalyzed to products in all slices). As a verification that the simulation is proceeding as expected, we can define the values of *V*_max_ and *K*_*M*_ to reasonably match the experimental values of free GluOx in the solution (therefore chosen to be 137 U/mg and 19 mM respectively). We choose a “mass” of enzyme in the simulation corresponding to the predicted mass of enzyme within the experimental hydrogel, 1.84 mg. As the experimental setup for the hydrogel samples effectively localizes the enzymes within the gel to ∼10% of the total sample length, we therefore scale this mass of enzyme by 10% such that it becomes evenly distributed over the full simulation length. We therefore achieve a final activity of 137 × 0.1 × 1.84 = 25.2 U across all slices of this solution state simulation. At a time *t*_0_, the simulation begins such that a given concentration of reactant occupies the full length of the model. The model is then allowed to proceed with a time step of 3 ms for 900,000 steps. The equivalent experimental signal is generated by monitoring the rate of change of concentration of products in the central slice of the model (position ∼1 cm, indicated by the red dot in Figure 8a) over a simulated time between 2000 and 2500 s and is fit using Michaelis–Menten kinetics. The predicted Michaelis–Menten kinetics for this simulation is shown in Figure 8b. This simulation predicts measured values of *V*_max_ and *K*_*M*_ of 137.9 U/mg and 19.8 mM respectively. This slight discrepancy between the measured and supplied values of of *V*_max_ and *K*_*M*_ is likely due to the finite time step employed by the simulation.

We now investigate the effects of enzyme localization in a hydrogel on the predicted values of of *V*_max_ and *K*_*M*_ by allowing an area at the far right of the 2 cm simulation length of depth 2 mm (10% of total length and therefore now containing the full 1.84 mg of enzyme) to define the hydrogel, within which we allow enzymatic catalysis to take place under Michaelis–Menten kinetics (shown schematically in Figure 8a). Detailed studies of diffusion of molecules through poly(vinyl alcohol) (PVA) hydrogels by Richbourg et al.^76^ suggest that at a volume fraction of 7.5% (similar to the 90 mg/mL hydrogels employed in the kinetics study), the diffusion of solutes with hydrodynamic radii between 0.5 and 7.5 nm through the hydrogel matrix is slowed by a factor of ∼0.5 in all instances. Inspired by this observation, we allow the diffusion coefficient in the hydrogel area of the model *D*_gel_ to be half the value of the diffusion coefficient of the rest of the model *D*_0_, also used in the solution state simulation described above. *D*_0_ is set to 4.275 × 10^–4^ m^2^ s^–1^ to allow the simulation to proceed over a reasonable time scale. We again define *V*_max_ and *K*_*M*_ to closely match the experimental values of free GluOx, 137 U/mg and 19 mM, respectively. At *t*_0_, the simulation now begins such that a given concentration of reactant occupies the full length of the model, except for the 2 mm depth occupied by the localized enzyme. Reactants can then diffuse into the area occupied by the localized enzyme and be catalyzed into products through Michaelis–Menten kinetics, which then diffuse out to be detected at the position of ∼1 cm as previously. These results are listed in Figure 8b. This simulation then predicts measured values of *V*_max_ and *K*_*M*_ of 138.8 U/mg and 23.6 mM, respectively. This demonstrates that while localization clearly causes the predicted values of *V*_max_ and *K*_*M*_ to deviate from the true values, weakly decreasing in the case of *V*_max_ and significantly increasing in the case of *K*_*M*_, it is still not enough to account for the experimentally measured signal.

The CD data presented then suggest that over time, the folded fraction of the enzyme in the hydrogel approaches 60%, regardless of enzyme concentration. This suggests that the activity of the hydrogel compared with free enzyme will reduce by a roughly equivalent amount and that the 24 h swelling period, which effectively reduces the enzyme concentration, is unlikely to affect this as it is shown to be enzyme concentration invariant. We can account for this effect within the simulation by decreasing the defined specific activity of the enzyme while preserving the value of *K*_*M*_. The results for a simulation beginning with localized enzyme with a *V*_max_ and *K*_*M*_ of 68 U/mg and 19 mM, respectively, are shown in Figure 8b. These yield predicted values for *V*_max_ and *K*_*M*_ of 68.5 U/mg and 22.7 mM respectively. This iteration more closely approaches the experimental data but still shows a measured value of *K*_*M*_ to be too low.

As a final iteration to account for this discrepancy in *K*_M_, we can define the values of *V*_max_ and *K*_*M*_ to now be 68 U/mg and 41 mM. The predicted signal from this simulation is shown in Figure 8b. Under these conditions, the simulated data now closely matches the experimental data and gives predicted values of *V*_max_ and *K*_*M*_ of 68.4 U/mg and 48.2 mM, respectively, and suggests that the true value of *V*_max_ and *K*_*M*_ of GluOx in the gel are close to the defined values of 68 U/mg and 41 mM. CD data suggest a folded fraction of 61% at long time after gelation, which will result in enzyme inactivation and therefore scale specific activity by an equivalent amount, and one therefore might expect a specific activity of ∼84 U/mg. As the experimentally determined value for specific activity is lower than this, this suggests that either 1.84 mg of enzyme in the hydrogel is a slight overestimate and the actual mass is slightly lower (∼1.49 mg) or that the experimentally derived activity of the free enzyme, 137.4 U/mg, does not scale by the 61% after gelation as suggested by the CD data but actually retains approximately 50%. This additional inactivation likely arises from the swelling of the hydrogel during the washing procedure, resulting in further enzyme unfolding, which is not performed during CD measurements. By considering the calculated volume ratio and measured specific activity, we can therefore say that a photochemically cross-linked all-enzyme GluOx hydrogel prepared at an initial concentration of 90 mg/mL and treated as described here has a volumetric productivity of ∼4000 U/cm^3^. These data therefore serve as an important proof of concept that photochemical cross-linking can be exploited to form functional hydrogels. This lays the foundation for a more detailed exploration of the links between mechanical stability, gel formation parameters such as enzyme concentration, cross-linking densicross-linking reaction rate, and hydrogel activity. Previous results, and classical polymer theory, dictate that a greater cross-linking density through increased building-block valency or concentration corresponds to increased mechanical stability;^31,35,46,77^ however, the associated reduction in mesh size results in hindered diffusion of products and substrates to the enzymes, resulting in lower activity.^28,31^ The balance of these important parameters for applications of these biomaterials, such as in biomedical devices or flow biocatalysis, will be the subject of future investigations. The longevity of all-enzyme hydrogels prepared through this method must also be considered, as previous studies using SpyTag/SpyCatcher chemistry have shown that the gels can remain active on the order of days.^78−81^

## Conclusions

In this study, we demonstrate that photochemical cross-linking of enzymes can produce functional all-enzyme hydrogels with tunable mechanical properties that retain the functionality of the enzyme. This approach is rapid, with gelation occurring on the order of minutes, and user-triggered due to the photochemical nature of the cross-linking. It allows for gels to be cast into desired shapes by employing simple surface-treated transparent molds, resulting in mechanically robust materials that require no modification to the initial enzyme and exhibit excellent volumetric productivity by allowing the enzyme to be both structural and functional. The current results suggest that the force exerted on the individual enzymes through the gelation process causes a degree of monomerization and enzyme unfolding, which in turn leads to inactivation of the enzyme. Enzyme monomerization is suggested to be the rate limiting step to gel formation, resulting in the unusual trend that the gel lag time increases with increasing enzyme concentration. By adopting a simple 1D diffusive model, we can account for the diffusion limitations of the Trinder assay and determine that upon gelation, the enzyme substrate affinity is reduced, reflected by an increase in *K*_*M*_ from 18.6 to 41 mM, and that 50% of the enzymatic activity remains. Future studies will consider alternate surface/volume ratio hydrogels and modulation of gelation forces through variation of illumination intensity as methods of tuning the enzymatic activities of the hydrogels. This work lays the foundation for photochemical cross-linking as a method of producing functional biocompatible materials for a host of potential uses.

## Supporting Information Available

Details of the various equations, derivations, and fitting parameters used in the manuscript; rheological characterizations not explicitly shown; experimental validations of enzyme hydrogel contents with respect to the folded state of the supplied enzyme; successful washing of unreacted components. The doi link for data is: https://doi.org/10.5518/1590 (PDF)
