# Supplementary material for: Nanomachine Networks: Functional All-Enzyme Hydrogels from Photochemical Cross-Linking of Glucose Oxidase
Source: Biomacromolecules. 2025 Jan 23;26(2):1195–206. doi: 10.1021/acs.biomac.4c01519 (PMC11815861; doi:10.1021/acs.biomac.4c01519)
Supplement: Supplementary file 1 — bm4c01519_si_001.pdf [file bm4c01519_si_001.pdf]

Nanomachine Networks: Functional All-Enzyme Hydrogels from Photochemical Cross-Linking of Glucose Oxidase– Supplementary

Harrison Laurent[1], David Brockwell[2], Lorna Dougan[1,2*]

1. School of Physics and Astronomy, University of Leeds, Leeds, UK, LS2 9JT
2. Astbury Centre for Structural Molecular Biology, University of Leeds, Leeds, UK, LS2 9JT

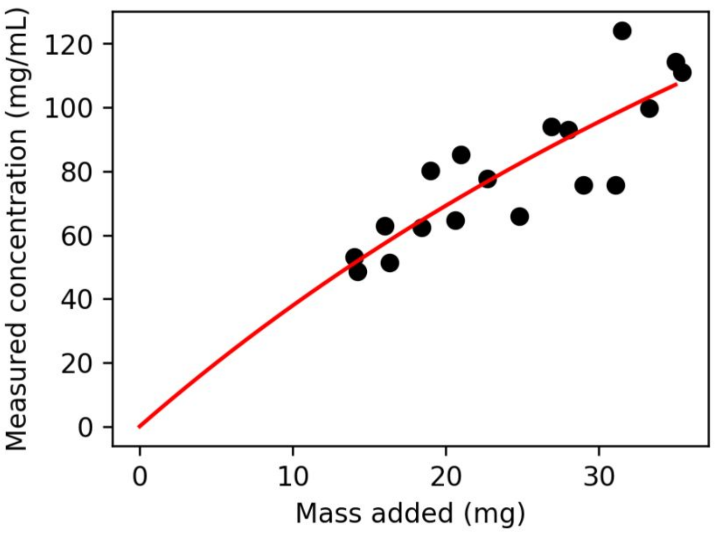

Fig S1. Predicted concentration in hydrogels based on UV-VIS measurements of diluted protein stocks as a function of mass of initially added GluOx. Raw data shown as black circles, fit shown in red.

$$[GluOx] = \frac{m_{GluOx}}{\alpha \times m_{GluOx} + \beta} \quad \text{(S1)}$$

Equation S1 derivation

The molar concentration of an enzyme $[e]$ in solution is defined as the ratio of the number of mols of the enzyme n_e over the total volume of the solution V_T

$$[e] = \frac{n_e}{V_T}$$

The number of mols of the enzyme is defined as the ratio of the mass of the enzyme m_e and the molar mass of the enzyme M_e

$$n_e = \frac{m_e}{M_e}$$

The total volume of the solution V_T is the sum of the volume occupied by the buffer V_B and the volume occupied by the enzyme V_e. Expressing V_e in terms of m_e and the density of the enzyme ρ_e, allows the following expression to be formed:

$$[e] = \frac{m_e}{M_e} \cdot \frac{1}{V_B + \frac{m_e}{\rho_e}} = \frac{m_e}{M_e \cdot V_B + \frac{M_e}{\rho_e} \cdot m_e} = \frac{m_e}{\alpha \cdot m_e + \beta}$$

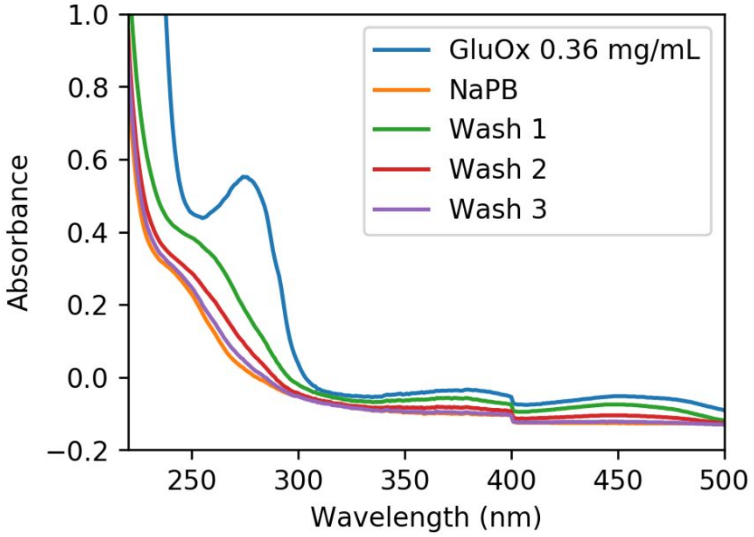

Fig S2. Absorption spectra of free GluOx in NaPB, the NaPB after the washing cycles as described in the Methods section, and unused PB. This data demonstrates any excess chemicals/unbound enzyme are gradually washed out of the hydrogel using this procedure.

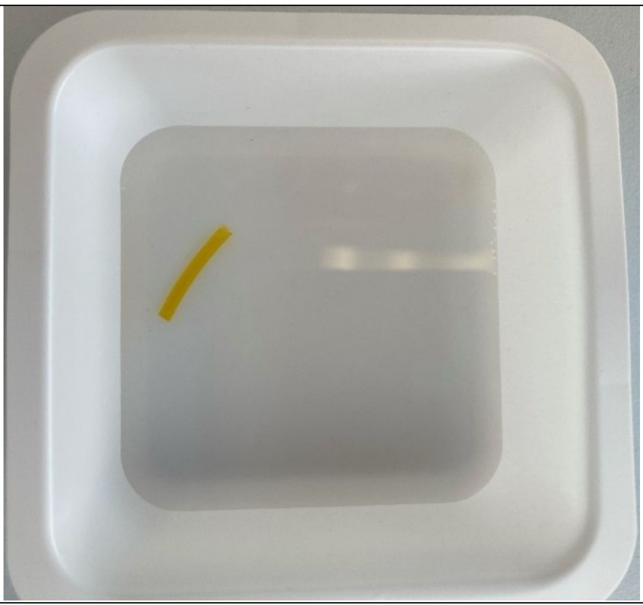

Fig S3. GluOx hydrogel prepared in a 2 mm diameter PTFE tube treated with Sigmacote

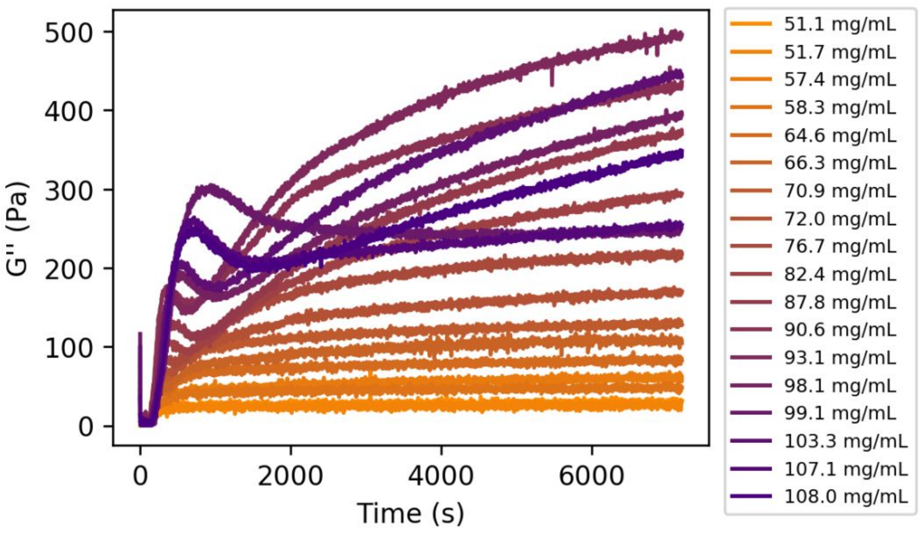

Fig S4. Time resolved loss modulus G'' as measured by oscillatory shear rheology of GluOx hydrogels at varying concentrations. Quoted concentrations correspond to enzyme volume fractions varying between 3.8 – 8.0 % assuming an enzyme density of 1.35 g/cm^3.

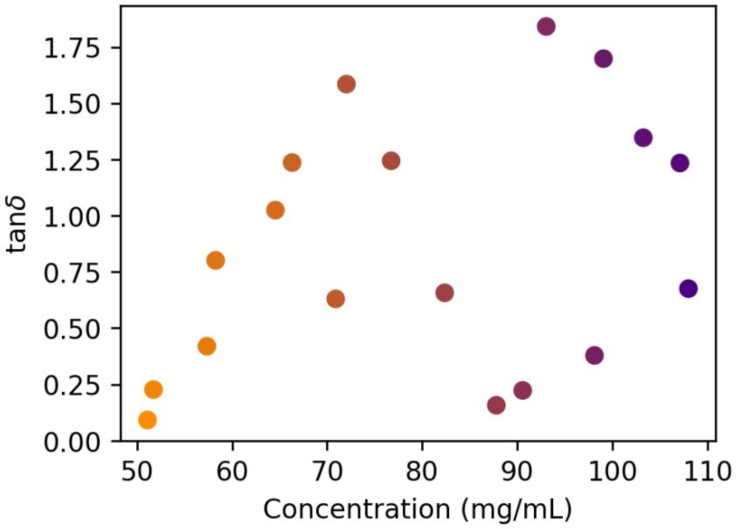

Fig S5. Values for $\tan\delta$ averaged over the first 60 s for GluOx hydrogel samples as a function of GluOx concentration prior to LED illumination

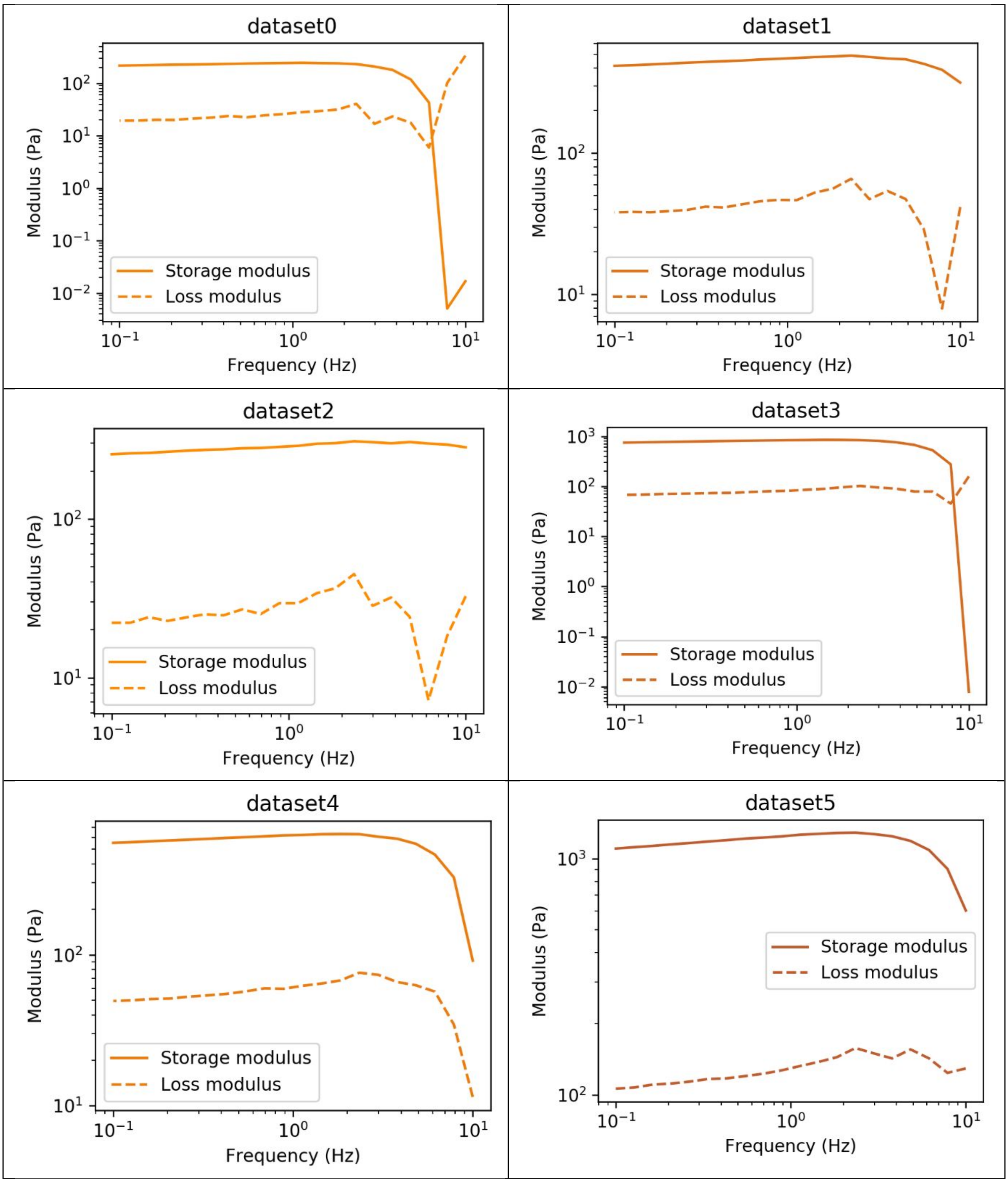
dataset0
dataset1
dataset2
dataset3
dataset4
dataset5
Storage modulus
Loss modulus
Modulus (Pa)
Frequency (Hz)
10^{-2}
10^{-1}
10^{0}
10^{1}
10^{2}
10^{3}

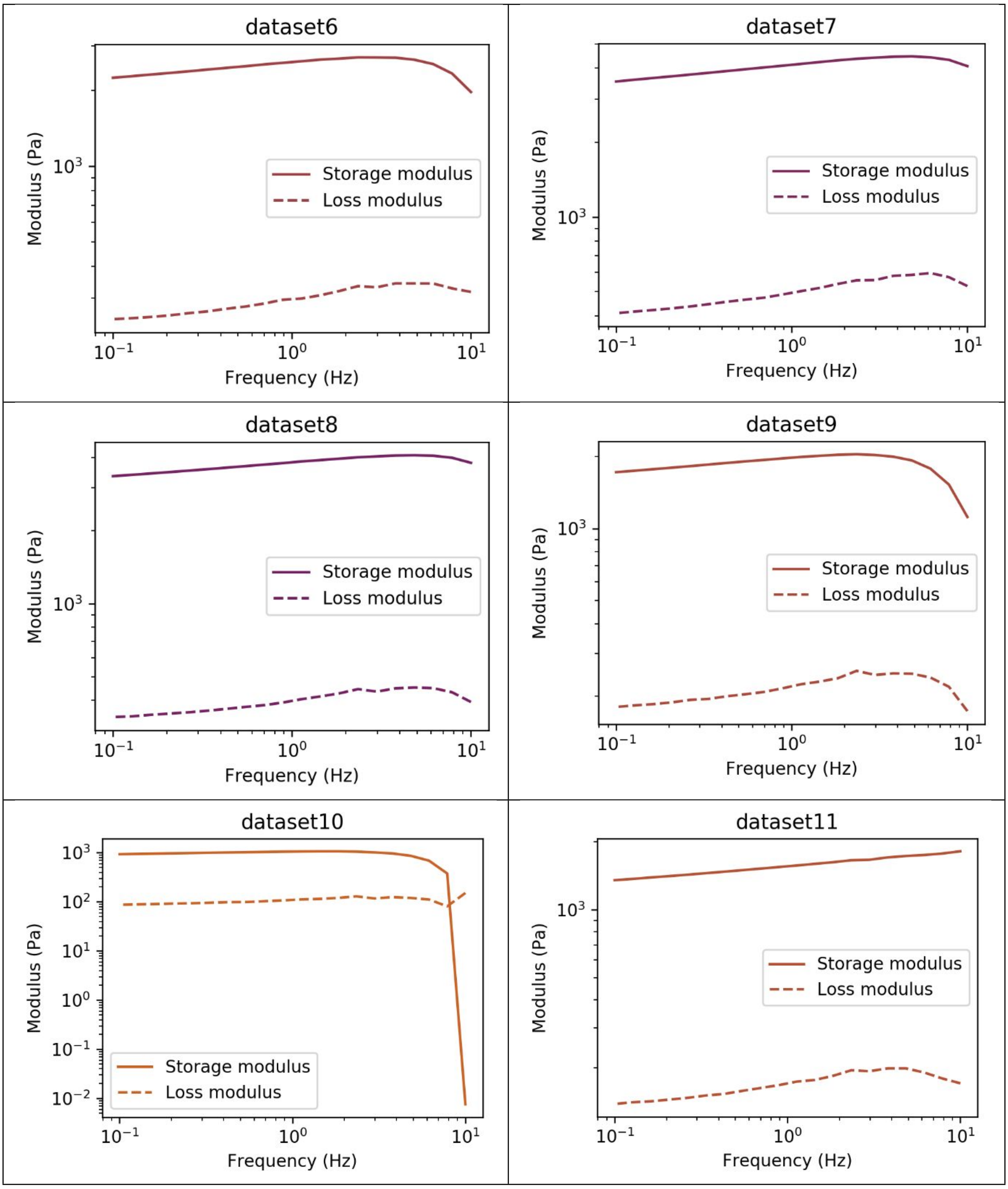
dataset6
Modulus (Pa)
10^3
Storage modulus
Loss modulus
Frequency (Hz)
10^{-1}
10^0
10^1
dataset7
dataset8
dataset9
dataset10
10^2
10^{-2}
dataset11

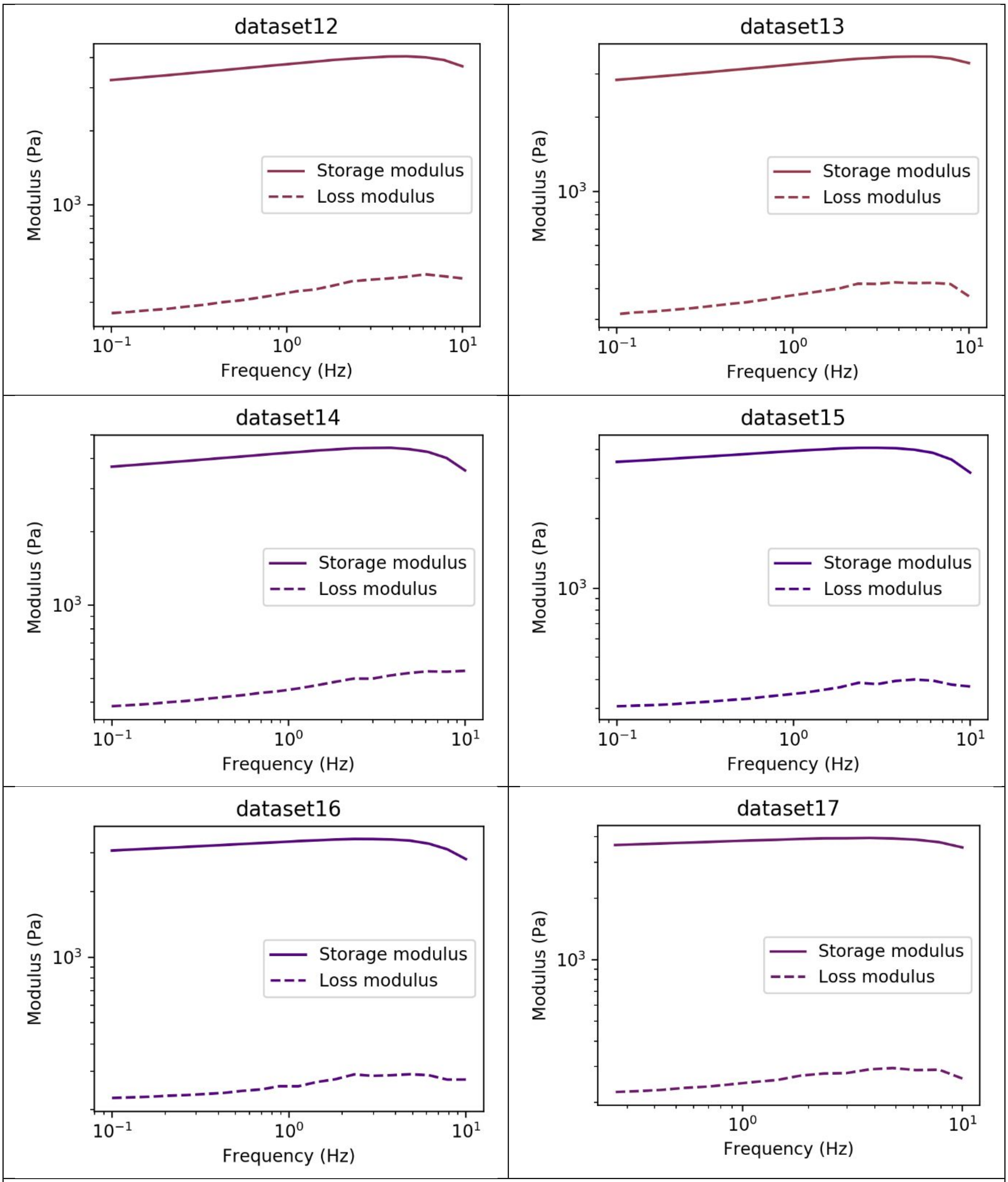

Fig S6. Frequency sweep data taken at $t = 7200\ s$ following gelation through photochemical cross-linking on custom rheology stage for GluOx hydrogels for the 18 investigated concentrations. Concentration of GluOx hydrogels investigated are listed in table S1.

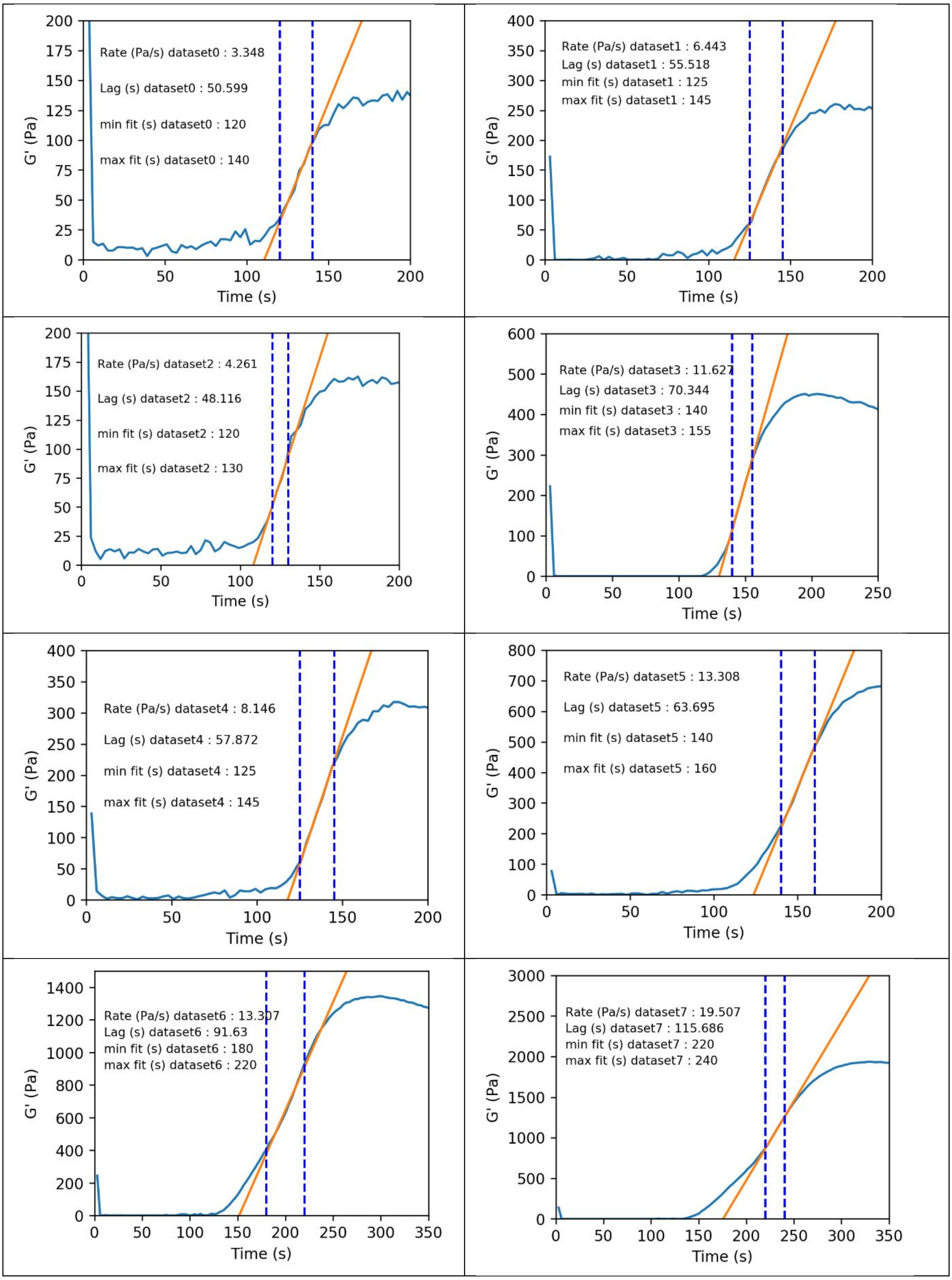

Rate (Pa/s) dataset0 : 3.348
Lag (s) dataset0 : 50.599
min fit (s) dataset0 : 120
max fit (s) dataset0 : 140
Rate (Pa/s) dataset1 : 6.443
Lag (s) dataset1 : 55.518
min fit (s) dataset1 : 125
max fit (s) dataset1 : 145
Rate (Pa/s) dataset2 : 4.261
Lag (s) dataset2 : 48.116
min fit (s) dataset2 : 120
max fit (s) dataset2 : 130
Rate (Pa/s) dataset3 : 11.627
Lag (s) dataset3 : 70.344
min fit (s) dataset3 : 140
max fit (s) dataset3 : 155
Rate (Pa/s) dataset4 : 8.146
Lag (s) dataset4 : 57.872
min fit (s) dataset4 : 125
max fit (s) dataset4 : 145
Rate (Pa/s) dataset5 : 13.308
Lag (s) dataset5 : 63.695
min fit (s) dataset5 : 140
max fit (s) dataset5 : 160
Rate (Pa/s) dataset6 : 13.307
Lag (s) dataset6 : 91.63
min fit (s) dataset6 : 180
max fit (s) dataset6 : 220
Rate (Pa/s) dataset7 : 19.507
Lag (s) dataset7 : 115.686
min fit (s) dataset7 : 220
max fit (s) dataset7 : 240
G' (Pa)
Time (s)

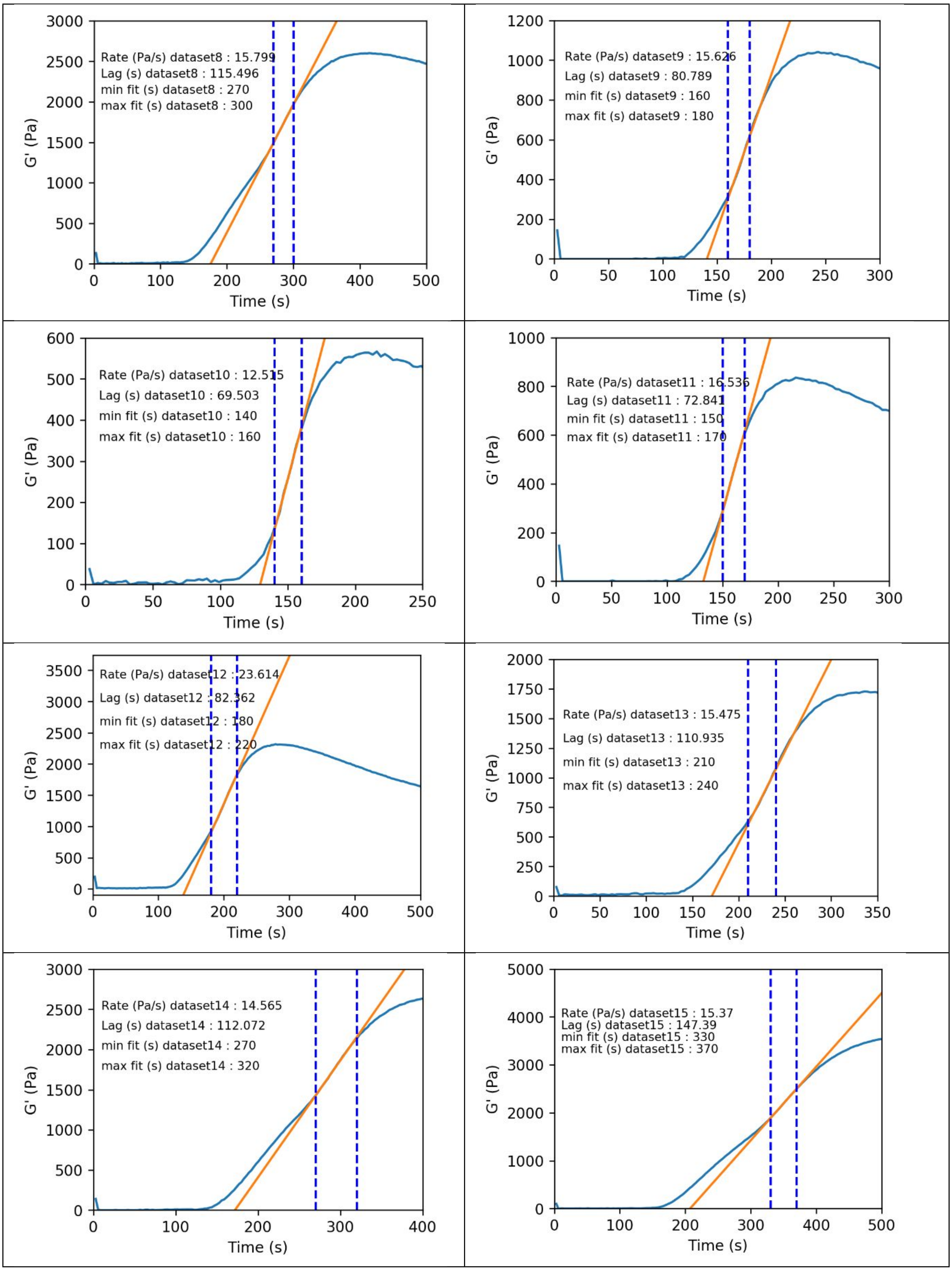
Rate (Pa/s) dataset8 : 15.799
Lag (s) dataset8 : 115.496
min fit (s) dataset8 : 270
max fit (s) dataset8 : 300
G' (Pa)
Time (s)
Rate (Pa/s) dataset9 : 15.626
Lag (s) dataset9 : 80.789
min fit (s) dataset9 : 160
max fit (s) dataset9 : 180
G' (Pa)
Time (s)
Rate (Pa/s) dataset10 : 12.515
Lag (s) dataset10 : 69.503
min fit (s) dataset10 : 140
max fit (s) dataset10 : 160
G' (Pa)
Time (s)
Rate (Pa/s) dataset11 : 16.536
Lag (s) dataset11 : 72.841
min fit (s) dataset11 : 150
max fit (s) dataset11 : 170
G' (Pa)
Time (s)
Rate (Pa/s) dataset12 : 23.614
Lag (s) dataset12 : 82.362
min fit (s) dataset12 : 180
max fit (s) dataset12 : 220
G' (Pa)
Time (s)
Rate (Pa/s) dataset13 : 15.475
Lag (s) dataset13 : 110.935
min fit (s) dataset13 : 210
max fit (s) dataset13 : 240
G' (Pa)
Time (s)
Rate (Pa/s) dataset14 : 14.565
Lag (s) dataset14 : 112.072
min fit (s) dataset14 : 270
max fit (s) dataset14 : 320
G' (Pa)
Time (s)
Rate (Pa/s) dataset15 : 15.37
Lag (s) dataset15 : 147.39
min fit (s) dataset15 : 330
max fit (s) dataset15 : 370
G' (Pa)
Time (s)

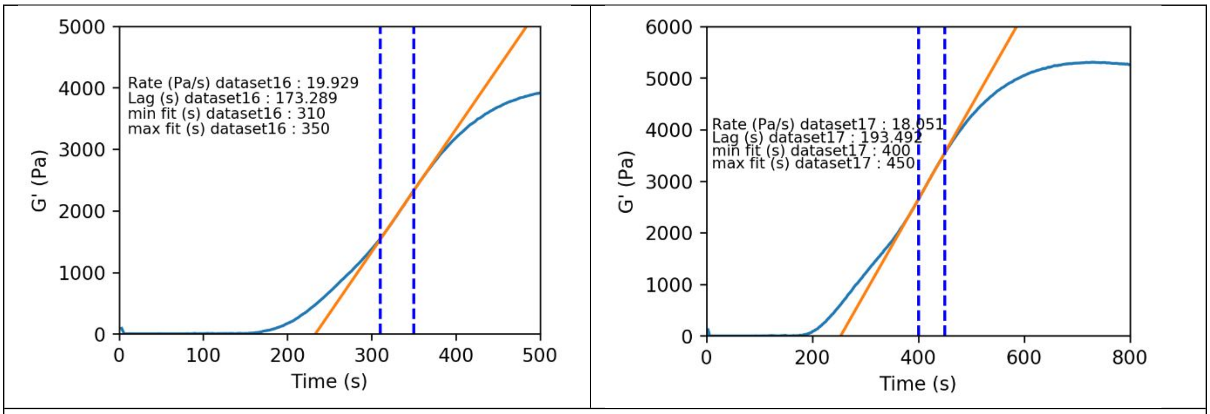

Fig S7. Calculation of k_{max} and t_{lag} from time resolved oscillatory shear rheology data for the 18 investigated GluOx hydrogels. Limits used for linear fitting shown as dashed blue lines. Raw data shown as solid blue line. Linear fit shown as orange line. Equation for linear fit given by $G'(t) = Gradient \times time + intercept$. Concentration of GluOx hydrogels investigated are listed in table S1.

**Name**	**Predicted concentration in gel** (mg/mL)
Dataset0	51.1
Dataset1	51.7
Dataset2	57.4
Dataset3	58.3
Dataset4	64.6
Dataset5	66.3
Dataset6	70.9
Dataset7	72
Dataset8	76.7
Dataset9	82.4
Dataset10	87.8
Dataset11	90.6
Dataset12	93.1
Dataset13	98.1
Dataset14	99.1
Dataset15	103.3
Dataset16	107.1
Dataset17	108

Table S1. Dataset names applied in frequency sweep data presented in Fig S5 and gel formation rate data shown in Fig S6 with corresponding calculated concentrations according to data presented in figure S1.

Empirical equation shown in figure 3(b) of main text, which describes the lag time of gelation as a function of concentration of GluOx in the hydrogels:

$$t_{lag} = Amplitude \times concentration^{power} \quad \text{(S2)}$$

Empirical equation shown in figure 3(c) of main text, which describes the maximum calculated gel formation rate as a function of concentration of GluOx in the hydrogels:

$$k_{max} = Amplitude \times (1 - \exp\left(-1 \times \frac{concentration - concentration_0}{constant}\right) \quad \text{(S3)}$$

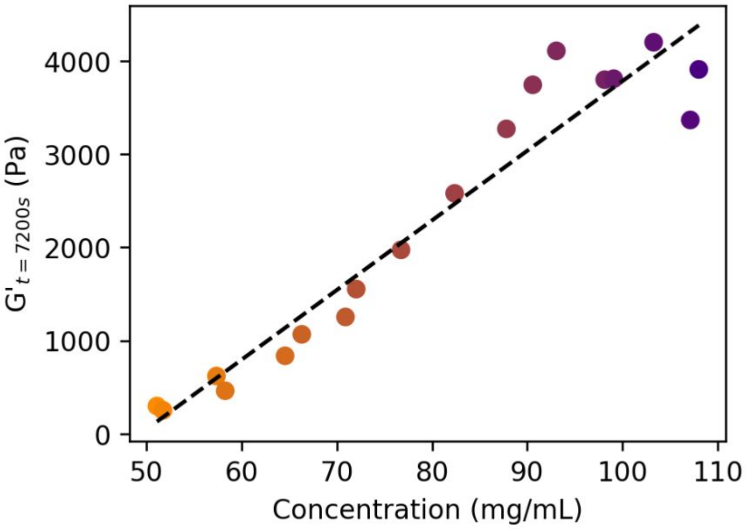

Figure S8. Linear fit to experimental storage modulus at $t = 7200\ s$ as a function of GluOx concentration in the hydrogels presented in Fig 4 of main text

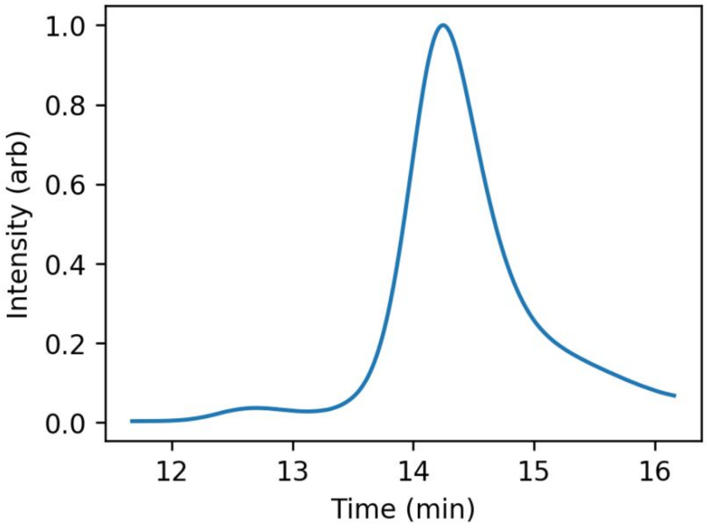

Figure S9. Size exclusion chromatography with multi-angle light scattering data to determine size of GluOx used in research. This data predicts that almost all measured protein has a predicted molecular weight of 137600 g/mol, which is close to the predicted weight of the dimer used in this research, 126546.8 g/mol.

**Parameter**	**50 mg/mL data**	**90 mg/mL data**
$C\ (s^{-1})$	0.093	0.036
$t_0\ (s)$	137.017	208.191
B_1	0.99	1.01
B_2	-1.0	-0.875
B_3	-0.265	-0.568
$\tau_1\ (s)$	66.932	345.494
$\tau_2\ (s)$	139.54	697.745
$\tau_3\ (s)$	2717.714	11347.848
$G_\infty\ (Pa)$	293.299	5413.793
$G_0\ (Pa)$	10.0	0.0

**Table S2.** Resultant fitting parameters to time resolved rheology data presented in figure 7 of main text using equation 7 in main text.

Note S1 - *Hydrogel Enzyme Kinetics Model Validation*

In order to validate the 1D enzyme hydrogel model, it is important that two criteria be separately satisfied:

1. As the diffusivity of the products/solutes increases to large values, and the diffusion limitation becomes effectively smaller, free enzyme like activity must be approached
2. If the simulation is allowed to proceed with the enzymes not located to a finite region of the model, but distributed over the entire model, this must again regain free enzyme like activity.

To test these points, a series of 'hydrogel state' simulations were set up, with the diffusion coefficient D_{gel} allowed to be equal to the diffusion coefficient outside the gel D_0. Values of K_M and V_{max} were chosen at 25 mM and 150 U/mg respectively. The mass of enzyme within the hydrogel region was chosen to be 5 mg. This therefore represents an activity of 750 U. As in the main text, the full length of the simulation was chosen to be 2 cm, with the hydrogel localised between 1.8-2.0 cm, and signal monitored at 1 cm. As this represents the hydrogel occupying 10% of the available simulation, another simulation was setup using equivalent values for D_0, K_M, and V_{max}, but allowing Michaelis-Menten kinetics over the full length with an appropriately scaled activity, hence the activity under these 'solution state' conditions was 75 U. The value for D_0 was then investigated for 5 values between 5.7×10^{-5} and 8.55×10^{-4} m^2s^{-1}. The results of these simulations are shown in figure S8. Here it is clear that as required, as the diffusion coefficient becomes sufficiently large such that diffusion is effectively no longer a limiting factor, free enzyme behaviour is approached, or the reaction is allowed to take place over the entirety of the simulation, free enzyme behaviour is recovered.

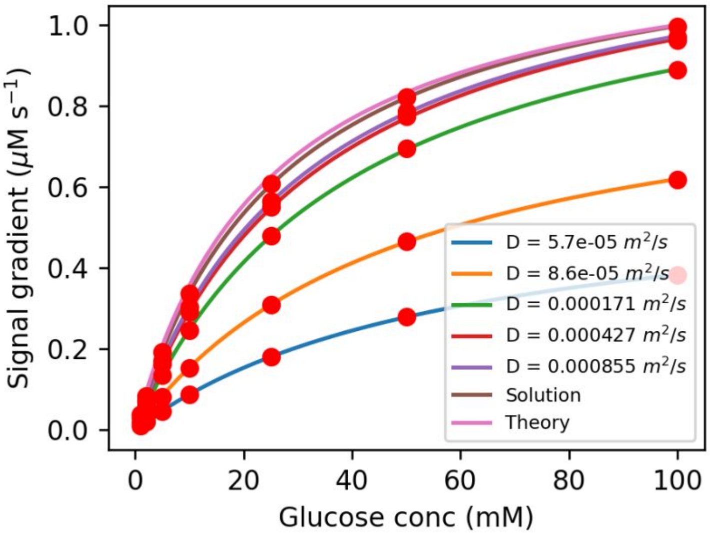

Figure S10. Calculated K_M and V_{max} parameters from 1D diffusion model as a function of bulk diffusion coefficient D_0. 5 different bulk diffusion coefficients tested, alongside the 'solution state' style simulation, where enzyme activity is allowed to take place over the entire simulation, and compared with the theoretical signal one would expect using a total enzyme activity of 75 U and a K_M of 25 mM.
